# LcCCL28-25, Derived from Piscine Chemokine, Exhibits Antimicrobial Activity against Gram-Negative and Gram-Positive Bacteria *In Vitro* and *In Vivo*

**DOI:** 10.1128/spectrum.02515-21

**Published:** 2022-05-26

**Authors:** Juanjuan Su, Haimeng Li, Jingyang Hu, Danni Wang, Fengchao Zhang, Zheng Fu, Feng Han

**Affiliations:** a School of Medicine and Pharmacy, Ocean University of China, Qingdao, China; b Laboratory for Marine Drugs and Bioproducts, Qingdao National Laboratory for Marine Science and Technology, Qingdao, China; c Key Laboratory of Marine Drugs, Ministry of Education, Qingdao, China; d Shandong Provincial Key Laboratory of Glycoscience and Glycotechnology, Qingdao, China; INTHERES

**Keywords:** bacterial resistance, antimicrobial peptide, antibacterial activity, negligible toxicity, *Staphylococcus aureus*

## Abstract

Antimicrobial peptides (AMPs) are currently recognized as potentially promising antibiotic substitutes. Fish are an important seawater/freshwater medicinal biological resource, and the antimicrobial peptides and proteins that are key components of their innate immune systems are potential candidates for the development of novel antibacterial agents. The rainbow trout Oncorhynchus mykiss chemokine CK11 (omCK11), classified in the C-C motif chemokine ligand 27/28 (CCL27/28) family, is the only CC-type chemokine reported to play a direct antibacterial role in the immune response; however, its antibacterial domain remains unknown. In this study, we analyzed the structure-activity relationship of omCK11 and identified the antibacterial C-terminal domain. Additionally, we performed structure-function analyses of CCL27/28 proteins from different, representative freshwater and seawater fishes, revealing their shared C-terminal antibacterial domains. Surprisingly, a synthesized cationic peptide (named lcCCL28-25), derived from the large yellow croaker Larimichthys crocea CCL28, exhibited broad-spectrum and the most acceptable bactericidal activity, as well as antibiofilm activity and negligible hemolytic and cytotoxic activity *in vitro*. Additionally, lcCCL28-25 conferred a protective effect in the thighs of neutropenic mice infected with Staphylococcus aureus. SYTOX green fluorescence and electron microscopy experiments revealed that lcCCL28-25 was capable of rapidly destroying the integrity and permeability of the bacterial cell membrane. Overall, this study aided in the advancement of antibacterial CC-type chemokine research and also suggested a new strategy for exploring novel AMPs. Additionally, the efficacy of lcCCL28-25 in *in vivo* antibacterial activity in a mammalian model revealed that this compound could be a promising agent for the development of peptide-based antibacterial therapeutics.

**IMPORTANCE** The primary function of chemokines has been described as recruiting and activating leukocytes to participate in the immune response. Some chemokines are also broad-spectrum antibacterial proteins in mammals. The Oncorhynchus mykiss chemokine CK11 (omCK11) is the first reported and currently the only CC-type antibacterial chemokine. The present study identified the antibacterial domain of omCK11. Structure-function analysis of various fish CCL27/28 proteins identified a novel antibacterial peptide (lcCCL28-25) from Larimichthys crocea CCL28 that exhibited broad-spectrum and the most acceptable bactericidal activity *in vitro*, as well as a protective effect in a Staphylococcus aureus infection mouse model. The antibacterial mechanisms included membrane disruption and permeation. This study advanced the field of antibacterial chemokine research in fish and also suggested a new strategy for exploring novel AMPs. The novel peptide lcCCL28-25 may prove to be an effective antibacterial agent.

## INTRODUCTION

Bacterial resistance is one of the most serious threats to global human health as declared by the World Health Organization (1). The emergence of multidrug-resistant bacteria and even so-called “superbugs” has been reported frequently in recent years (2). Therefore, it is critical to develop antibacterial drugs that make the development of resistance difficult.

Innate immunity mainly relies on the production of short peptides or proteins with antibacterial activity that serve as the initial line of defense against pathogenic microorganisms (3, 4). Natural antimicrobial peptides (AMPs) are a class of alkaline polypeptides with antibacterial activity produced by induction in the body and found in all organisms (5–7). With their excellent characteristics of rapid sterilization, low toxicity and side effects, low immunogenicity, and broad-spectrum activity without drug resistance, AMPs are widely recognized as an ideal alternative to antibiotics, providing mankind with new opportunities to combat drug-resistant microorganisms (5, 8).

Chemokines are a subclass of cytokines made of small molecules typically containing ~70 to 100 amino acids (9, 10). In addition to their chemotactic signaling activity, some mammalian chemokines are also broad-spectrum antibacterial proteins known as kinocidins, such as human CXCL1 (11), CXCL4 (12, 13), CXCL6 (14), CXCL7 (15) CXCL8 (11), CXCL9 (16), and CXCL14 (17) of the C-X-C motif chemokine ligand (CXCL) family, CCL2, CCL5, and CCL20 of the C-C motif chemokine ligand (CCL) family (11, 15), etc.

Due to a large number of genomic replication events and the fact that chemokines evolve more rapidly than other immune genes, it is difficult to establish true homology between fish and mammalian chemokines (18–20). Numerous fish chemokines have been found in recent years, and their roles overlap almost entirely with those of lymphocytes. Apart from recruiting and activating leukocytes to act on the site of infection and participate in the immune response (21, 22), fish chemokines also play important roles in stress response (23), tentacle development (24), embryo development and angiogenesis, etc. (25, 26). Unfortunately, the study of antimicrobial chemokines found in fish is still in its infancy. Rainbow trout Oncorhynchus mykiss chemokine CK11 (omCK11), which is classified as a CCL27/28 chemokine, is the first and currently the only CC-type fish chemokine reported to have antimicrobial activity and is capable of significantly inhibiting the growth of Lactococcus garvieae, Aeromonas salmonicida, Yersinia ruckeri, and other Gram-negative and -positive pathogens, as well as the parasite Ichthyophthirius multifiliis (27). However, the antimicrobial domain within the CK11 molecule has not been identified. Effective interception and modification of antibacterial core peptides is an effective way to overcome the disadvantages of high molecular weight and high immunogenicity of natural chemokines, as well as a new strategy for obtaining new antibacterial peptides.

In this study, we analyzed omCK11 and verified that its C-terminal α-helix structure was the antibacterial domain. Additionally, structural comparisons of CCL27/28 proteins from six representative fish species revealed that although these proteins have low homology, their C-terminal regions share cationic and amphiphilic commonalities. Antibacterial activity assays revealed that the C-terminal region of CCL28 of the large yellow croaker (Larimichthys crocea), named lcCCL28-25, exhibited the highest antibacterial activity. Overall, this study advanced the field of research into the antibacterial activity of CC-type chemokines and also suggested a new strategy for exploring novel AMPs. The novel peptide lcCCL28-25 may also prove to be a promising antibacterial agent.

## RESULTS

### Structural features of the proteins and analysis of their antimicrobial segments.

**FIG 1:**
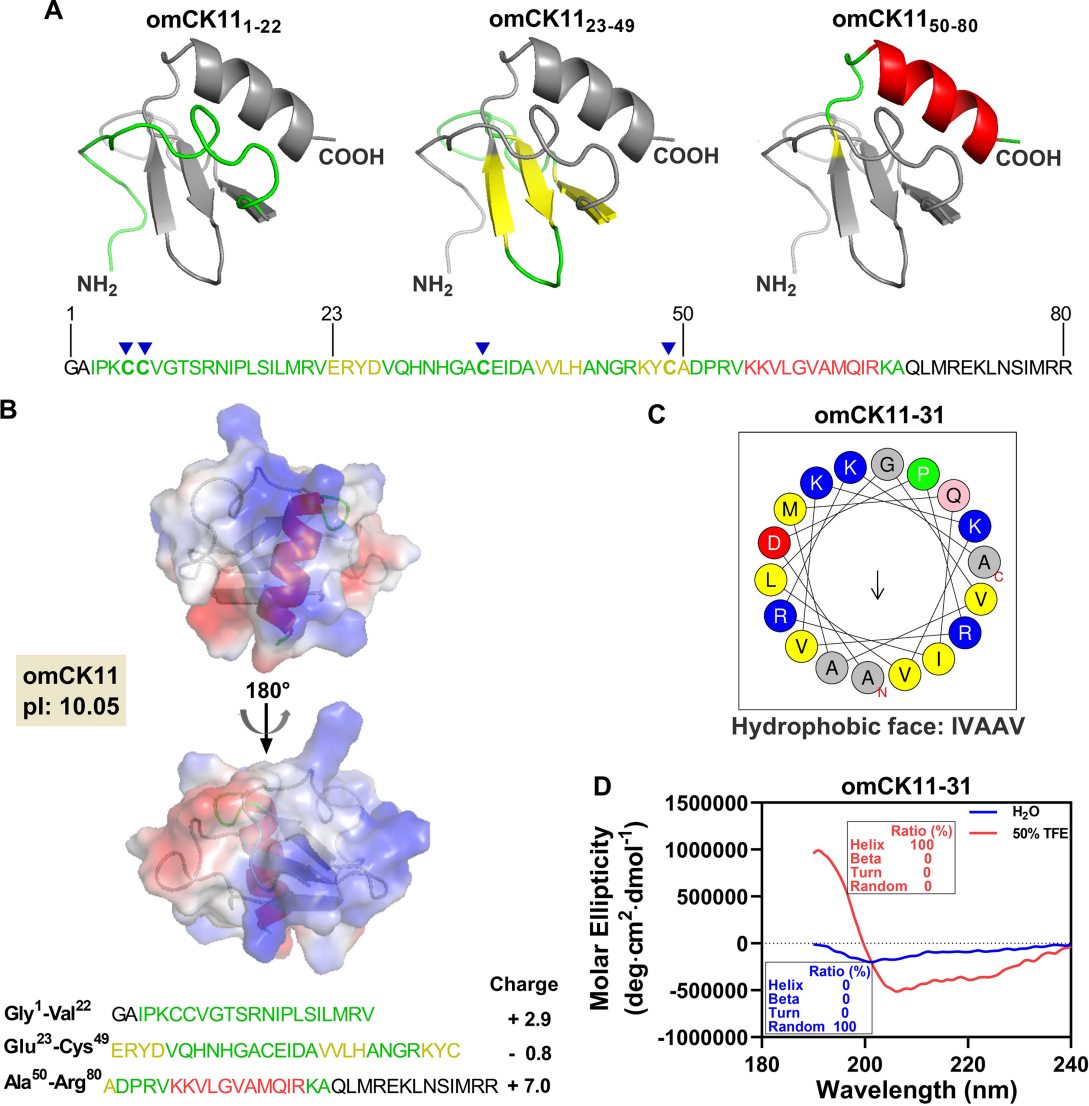
Structural features of O. mykiss CK11 (omCK11). (A) Three-dimensional model of omCK11. Structures in green, yellow, and red represent the N-terminal random coil region (Gly^1^–Val^22^), the middle 3-stranded antiparallel β-sheet region (Glu^23^–Ala^50^), and the C-terminal α-helix region (Asp^51^–Arg^80^). (B) Electrostatic potential surface maps of omCK11 in which areas with positive charges are shown in blue and negative charges in red. (C) Helical wheel plot of the omCK11-31 peptide. The yellow background points to the hydrophobic residues, F, L, M, I and V, the blue to the positively charged hydrophilic residues, K and R, and the red to the negatively charged hydrophilic residue (D). The light green represents the proline (P), the pink represents the asparagine (N) and glutamine (Q), and the gray represents glycine (G) and alanine (A). (D) CD spectroscopy of omCK11-31 (200 μg/mL) in water and 50% TFE solution.

The classical natural host defense proteins share several structural characteristics, including an α-helix, disulfide bridges, and a high proportion of cationic amino acids (17, 28). These distinct structural components may be great candidates for the designing of novel AMPs. The CK11 chemokine protein from rainbow trout (O. mykiss), omCK11, was the first chemokine with antimicrobial activity to be reported in fish (27). Through homology modeling, a three-dimensional model of omCK11 was predicted. omCK11 features a structure that is typical of mammalian chemokines, consisting of an N-terminal random coil (Gly^1^ to Val^22^), a core structure composed of three antiparallel β-strands (Glu^23^ to Ala^50^), and a C-terminal α-helix (Asp^51^ to Arg^80^) (Fig. 1A).

Electrostatic potential maps suggested that the surface of omCK11 was cationic (theoretical isoelectric point [pI] of 10.05) (Fig. 1B), owing to the presence of six lysines and nine arginines within its peptide sequence (Fig. 1A). Notably, the surface charge distribution of omCK11 was not uniform, but had a particularly dense distribution of positively charged amino acids in the C-terminal region, including four lysines and five arginines (Fig. 1A). Additionally, the C-terminal segment of the modeling sequence had a net charge of 7.0 (Fig. 1B). In comparison, the N-terminal and intermediate segments of omCK11 exhibited lower net charges (Fig. 1B). Moreover, helical wheel analysis revealed that the C-terminal segment of omCK11 was an amphiphilic peptide (Fig. 1C). Above all, the C-terminal peptide of omCK11 was a cationic amphiphilic peptide that conformed to the characteristics typical of the majority of natural AMPs. Therefore, the C-terminal peptide may represent the antibacterial domain of omCK11. This peptide segment contains 31 amino acids and is hence referred to as omCK11-31. Additionally, circular dichroism (CD) spectroscopy demonstrated that omCK11-31 maintained a 100% random coil structure in water but formed a 100% helix structure in a 50% trifluoroethanol (TFE) solution, which simulates a hydrophobic membrane environment (Fig. 1D), confirming the accuracy of the specific cationic and amphiphilic α-helical structure of omCK11-31. These features implied that omCK11-31 could be a highly potent AMP.

**FIG 2:**
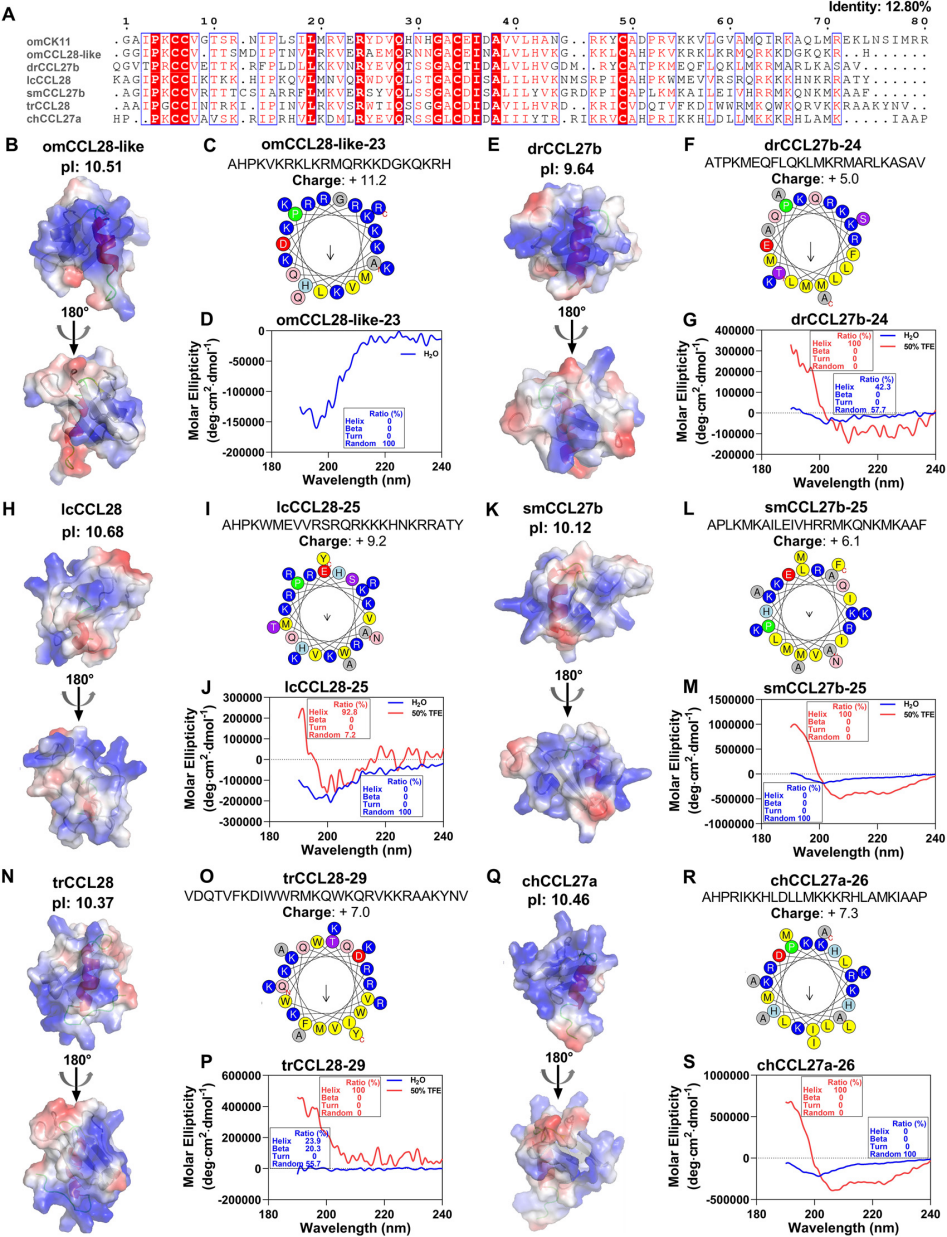
Protein sequence alignment, electrostatic potential surface diagram, and helical wheel plot analysis. (A) Protein sequence alignment of O. mykiss CK11 (omCK11; GenBank accession no. CDQ77591.1), *O. mykiss* CCL28-like (omCCL28-like; GenBank accession no. CDQ62061.1), D. rerio CCL27b (drCCL27b; GenBank accession no. NP_001121740.1), L. crocea CCL28 (lcCCL28; GenBank accession no. XP_010731843.1), S. maximus CCL27b (smCCL27b; GenBank accession no. XP_035496366.1), T. rubripes CCL28 (trCCL28; GenBank accession no. NP_001266955.1), and C. harengus CCL27a (chCCL27a; GenBank accession no. XP_012669911.2). Red background represents the same amino acid residues, and blue frames indicate amino acid residues with identities of >70%. (B, E, H, K, N, and Q) Electrostatic potential surface diagrams of proteins. Areas with positive charges are indicated in blue, and negative charges in red. (C, F, I, L, O, and R) Helical wheel plots of the C-terminal segments. The hydrophobic residues, F, L, M, I, V, Y, and W are in a yellow background, the positively charged hydrophilic residues, K and R, in blue, and the negatively charged hydrophilic residues, D and E in red. Light green represents proline (P), light blue represents histidine (H), pink represents asparagine (N) and glutamine (Q), purple represents serine (S) and threonine (T), and gray represents glycine (G) and alanine (A). (D, G, J, M, P, and S) CD spectroscopy of the C-terminal peptides (200 μg/mL) in water and 50% TFE solution. (C, D, F, G, I, J, L, M, O, P, R, and S) omCCL28-like-23 (C, D), drCCL27b-24 (F, G), lcCCL28-25 (I, J), smCCL27b-25 (L, M), trCCL28-29 (O, P), and chCCL27a-26 (R, S).

In this study, we performed a comparative analysis of CCL27/28 family proteins in representative freshwater and seawater fishes. Further investigation was carried out on O. mykiss CCL28-like (omCCL28-like), zebrafish Danio rerio CCL27b (drCCL27b), L. crocea CCL28 (lcCCL28), turbot Scophthalmus maximus CCL27b (smCCL27b), tiger puffer Takifugu rubripes CCL28 (trCCL28), and Atlantic herring Clupea harengus CCL27a (chCCL27a). As shown by the alignment in Fig. 2A, the sequence similarity of CCL27/28 between various fishes was very low (identity of 12.80%), with their C-terminal regions being particularly unconserved. Subsequent analysis of the structures and surface electrostatic potentials of CCL27/28 proteins from different fishes revealed that the secondary structure characteristics of CCL27/28 were similar to those of omCK11 (Fig. 2B, E, H, K, N, and Q), which was consistent with the typical characteristics of traditional CCL-type chemokines. Moreover, their surfaces were all cationic (pI of >7.0) (Fig. 2B, E, H, K, N, and Q) and the net charges of their C-terminal segments were all greater than 5.0 (Fig. 2C, F, I, L, O, and R). Additionally, helical wheel analysis demonstrated the amphiphilic characteristics of most of the C-terminal segments (Fig. 2C, F, I, L, O, and R). Following that, all C-terminal peptides were synthesized and assigned the names omCCL28-like-23, drCCL27b-24, lcCCL28-25, smCCL27b-25, trCCL28-29, and chCCL27a-26. Their high-performance liquid chromatography (HPLC) and mass spectrometry (MS) analysis results are shown in Fig. S1 to S14 in the supplemental material. Furthermore, the specific cationic and amphiphilic α-helical structures of the C-terminal segments, except for that of omCCL28-like, were verified by CD spectroscopy measurements in water and in 50% TFE solution (Fig. 2D, G, J, M, P, and S). Because the synthesized omCCL28-like-23 was insoluble in a 50% TFE solution (data not shown), its CD spectroscopy was not determined in this research.

### Antibacterial activities of different C-terminal peptides *in vitro*.

The antimicrobial activities of omCK11-31 and other peptides were evaluated against five Gram-negative bacteria (Escherichia coli strain ATCC 25922, Klebsiella pneumoniae strain CMCC46117, Aeromonas hydrophila strain ATCC 7966, Pseudomonas aeruginosa strain PAO1, and Pseudomonas aeruginosa strain FRD1) and five Gram-positive bacteria (Staphylococcus aureus strain ATCC 25923, Streptococcus agalactiae strain ATCC 13813, Streptococcus pneumoniae strain ATCC 49619, methicillin-resistant S. aureus strain ATCC 43300, and methicillin-resistant S. aureus standard strain USA300) (Table 1). MIC assays confirmed that omCK11-31 was the domain of omCK11 with an antibacterial function, exhibiting broad-spectrum antibacterial activity against both Gram-negative and Gram-positive bacteria. The other six peptides exhibited similar broad-spectrum antimicrobial activities. For Gram-negative bacteria, the MICs of all investigated peptides for E. coli ATCC 25922, P. aeruginosa PAO1, and P. aeruginosa FRD1 ranged between 6.25 and 50 μg/mL. All investigated peptides exhibited poor antibacterial activity against K. pneumoniae CMCC46117 and A. hydrophila ATCC 7966 (most MIC values were greater than 50 μg/mL).

**TABLE 1:** Peptide MICs determined in MHB

Bacterial strain	MIC (μg/mL) of:						
	omCK11-31	omCCL28-like-23	drCCL27b-24	lcCCL28-25	smCCL27b-25	trCCL28-29	chCCL27a-26
Gram-negative bacteria							
Escherichia coli ATCC 25922	12.50	12.50	25.00	6.25	50.00	12.50	25.00
Klebsiella pneumoniae CMCC46117	25.00	>50.00	>50.00	25.00	>50.00	25.00	>50.00
Aeromonas hydrophila ATCC 7966	>50.00	>50.00	>50.00	>50.00	>50.00	25.00	>50.00
Pseudomonas aeruginosa PAO1	25.00	25.00	25.00	12.50	50.00	25.00	25.00
P. aeruginosa FRD1	12.50	25.00	25.00	12.50	25.00	25.00	25.00

Gram-positive bacteria							
Staphylococcus aureus ATCC 25923	12.50	12.50	25.00	6.25	25.00	12.50	12.50
Streptococcus agalactiae ATCC 13813	12.50	12.50	25.00	6.25	25.00	12.50	25.00
Streptococcus pneumoniae ATCC 49619	12.50	25.00	25.00	6.25	25.00	25.00	25.00
Methicillin-resistant S. aureus ATCC 43300	12.50	25.00	25.00	12.50	>50.00	12.50	25.00
Methicillin-resistant S. aureus USA300	12.50	12.50	25.00	6.25	25.00	12.5	25.00

All of the peptides studied had much greater antibacterial impacts against Gram-positive bacteria than they did against Gram-negative bacteria. Except for smCCL27b-25, the MICs of the tested peptides against S. aureus ATCC 25923, S. agalactiae ATCC 13813, S. pneumoniae ATCC 49619, and methicillin-resistant S. aureus ATCC 43300 ranged between 6.25 and 25 μg/mL. Notably, of the seven peptides tested, lcCCL28-25, derived from L. crocea, exhibited the strongest antibacterial activity. Therefore, lcCCL28-25 was selected for further research.

### Kill-curve kinetics of lcCCL28-25.

**FIG 3:**
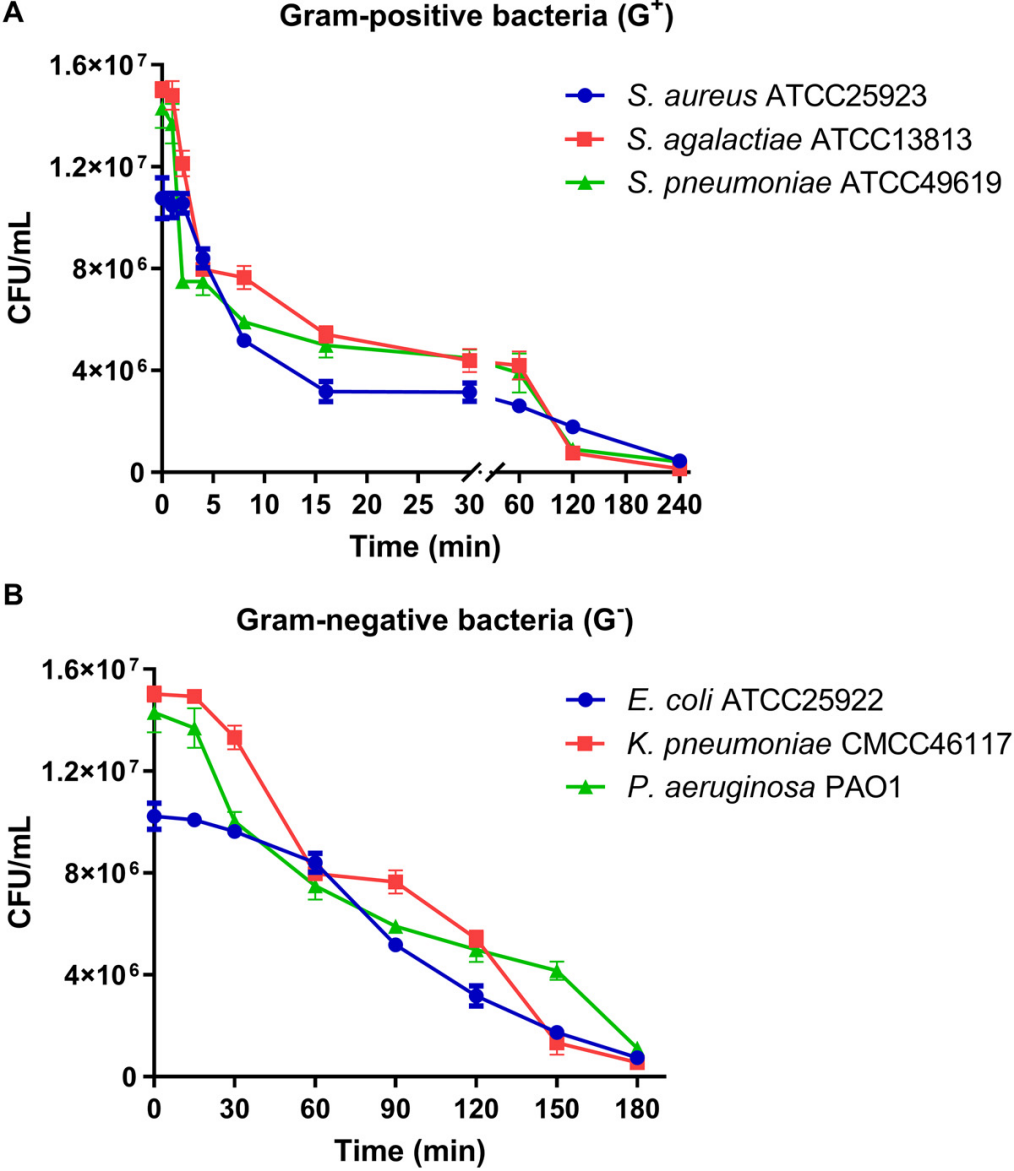
Time-kill curves of lcCCL28-25 (4× MIC) against Gram-positive bacteria (A) and Gram-negative bacteria (B). Data are presented as mean values ± standard deviations.

Time-kill assays were performed at 4× MICs of lcCCL28-25. The results demonstrated the broad-spectrum antibacterial activity of lcCCL28-25 against Gram-negative and Gram-positive bacteria (Fig. 3). The antibacterial activity of lcCCL28-25 against Gram-positive bacteria was more rapid than that against Gram-negative bacteria. Under 4× MIC conditions, the CFU counts of Gram-positive bacteria could be reduced by more than half within 10 min (Fig. 3A), whereas the CFU counts of Gram-negative bacteria could be reduced by the same amount after more than 1 h (Fig. 3B).

### Toxicity of lcCCL28-25 *in vitro*.

**FIG 4:**
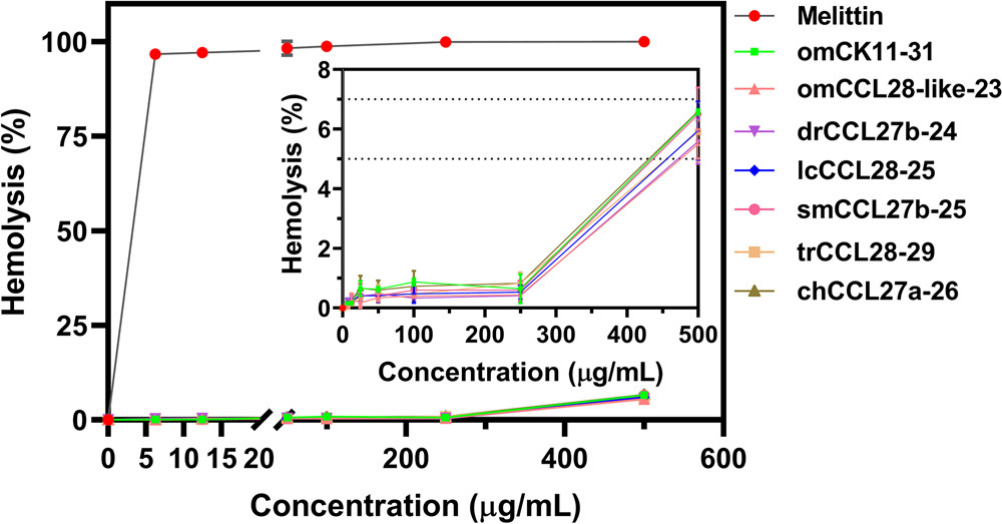
Hemolytic activities of peptides against human red blood cells (hRBCs). The absorbance value (*A*_540_) for 0% hemolysis was determined using phosphate-buffered saline (PBS) (*A*_PBS_), while 100% hemolysis was established using 0.1% (vol/vol) Triton X-100 (*A*_Triton_). Percentages of hemolysis were calculated as follows: % hemolysis = [(*A*_sample_ − *A*_PBS_)/(*A*_Triton_ − *A*_PBS_)] × 100. Each measurement was performed in triplicate. Data are presented as mean values ± standard deviations.

**FIG 5:**
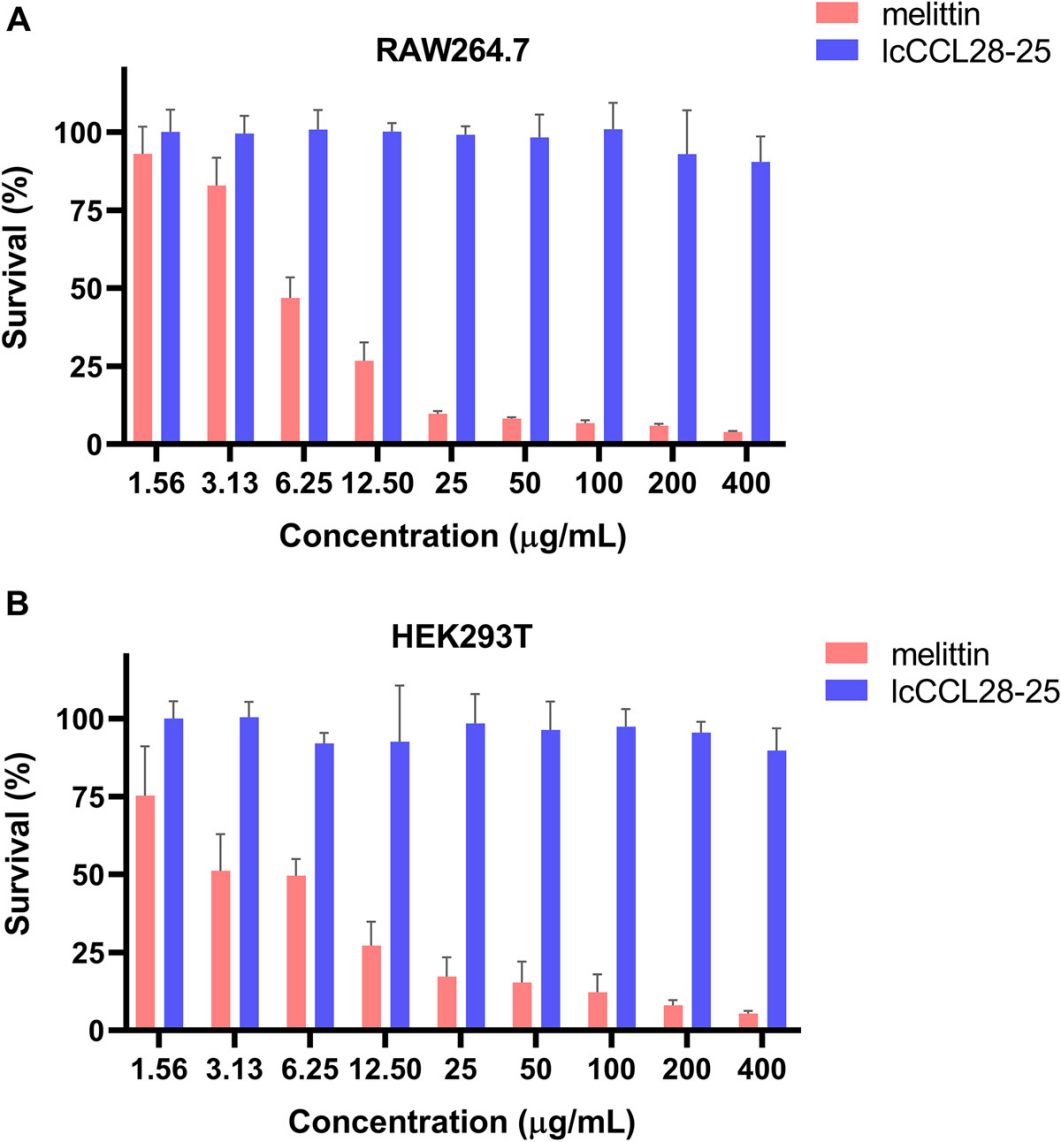
Cytotoxicity of lcCCL28-25 toward RAW264.7 cells (murine monocyte/macrophage cells lines) (A) and HEK293T cells (human embryonic kidney cells) (B). The MTT assay was used. PBS was used to establish the value for 100% survival. Each measurement was performed in triplicate. Data are presented as mean values ± standard deviations.

The toxicities of lcCCL28-25 and six other peptides were evaluated by analyzing their hemolytic activities against human red blood cells (hRBCs). Compared with that of melittin, all of these seven peptides demonstrated extremely low hemolytic activities, with membrane lytic activities of <7% at the maximum measured concentration of 500 μg/mL (Fig. 4). Additionally, the MTT [3-(4,5-dimethyl-2-thiazolyl)-2,5-diphenyl-2H-tetrazolium bromide] assay results showed that lcCCL28-25 displayed low toxicities toward RAW264.7 (Fig. 5A) and HEK293T cells (Fig. 5B). No significant inhibition of cell viability was observed even at a high concentration of 400 μg/mL (Fig. 5), indicating that lcCCL28-25 had weak toxicity toward mammalian cells.

### Biofilm inhibition and eradication activities of peptides.

**FIG 6:**
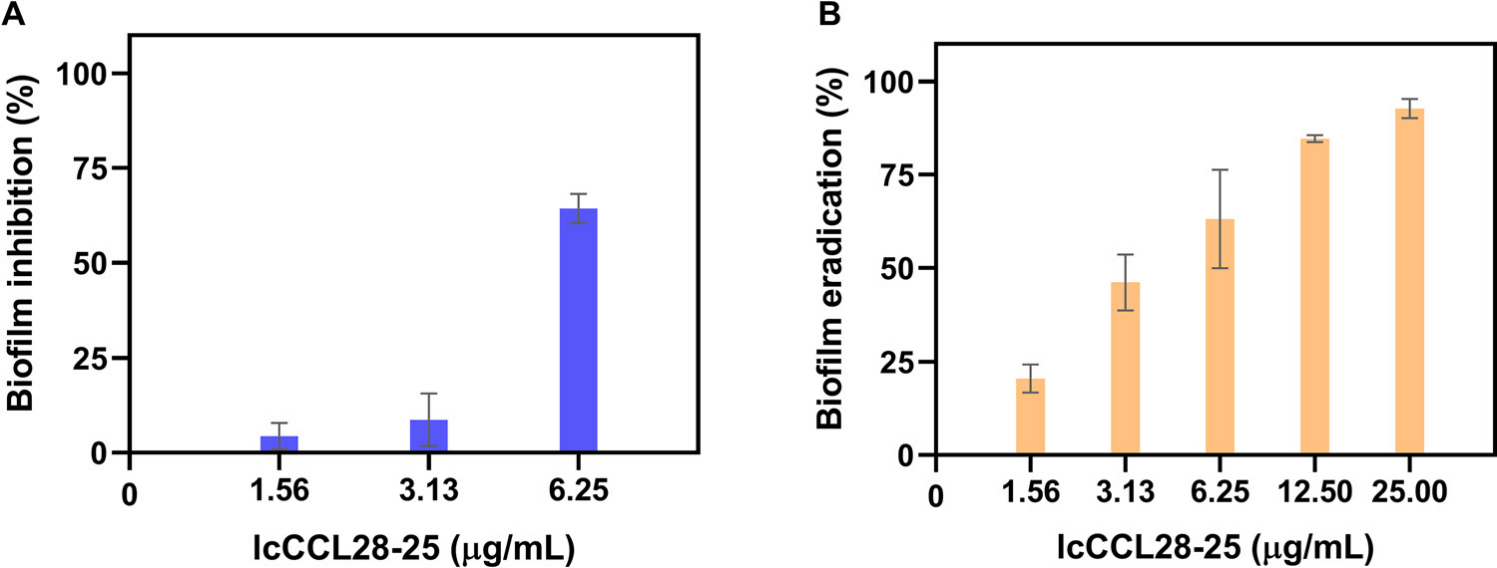
Inhibition (A) and eradication (B) activities of lcCCL28-25 on P. aeruginosa FRD1 biofilm. The value for 0% inhibition or eradication was established without peptide. Each measurement was performed in triplicate. Data are presented as mean values ± standard deviations.

P. aeruginosa is a well-known biofilm-forming pathogen. The clinical mucoid strain FRD1 was used to determine the effects of all these seven peptides on biofilm formation (inhibition effect) and clearance (eradication effect). As shown by the results in Fig. 6A, lcCCL28-25 reduced biofilm formation by ~64% and omCK11-31 reduced it by ~45% under sub-MIC conditions (6.25 μg/mL, 0.5× MIC). However, the inhibitory rates of the other five peptides were all less than 15% at the same concentration (Fig. S15A). The results in Fig. 6B show the eradication effect of lcCCL28-25 on a 24-h mature biofilm. The concentration of lcCCL28-25 required to eradicate ~50% of biofilm was 3.13 μg/mL. The biofilm eradication rates reached more than 80% at 12.5 μg/mL of lcCCL28-25 and above 90% at 25 μg/mL (Fig. 6B). The biofilm eradication effect of omCK11-31 was similar to that of lcCCL28-25, and both were superior to the other five peptides (Fig. 6B and Fig. S15B). Increasing the peptide concentration resulted in increased biofilm eradication, demonstrating the concentration-dependent eradication effects of all of these seven peptides on biofilm (Fig. 6B and Fig. S15B). Overall, lcCCL28-25 had considerable biofilm inhibition and eradication capacities.

### Salt and serum stability.

In order to evaluate the effects of salts and mouse serum on the antimicrobial activity of lcCCL28-25 against S. aureus USA300, the MIC was tested under conditions of the physiological salt concentration or different concentrations of mouse serum. As shown by the results in Table 2, combinations of lcCCL28-25 with KCl, NH_4_Cl, and MgCl_2_ displayed activities similar to that of only lcCCL28-25, and combinations of lcCCL28-25 with NaCl and CaCl_2_ presented 4-fold and 2-fold decreases in activity, respectively. Consistent with the results for melittin, the antimicrobial activity of lcCCL28-25 against S. aureus USA300 was reduced 4-fold, 8-fold, and 32-fold under 10%, 20%, and 40% serum conditions, respectively.

**TABLE 2:** MIC values of peptides in the presence of physiological salts and mouse serum against Staphylococcus aureus USA300

Peptide	MIC (μg/mL) in^*a*^:								
	Control	150 mM NaCl	4.5 mM KCl	6 μM NH_4_Cl	1 mM MgCl_2_	2.5 mM CaCl_2_	MS at concn (%) of:		
							10	20	40
lcCCL28-25	6.25	25.00	6.25	6.25	6.25	12.50	25.00	50.00	200.00
Melittin	6.25	6.25	6.25	6.25	6.25	6.25	25.00	50.00	200.00

a The control was bacteria treated with peptide only. MS, mouse serum.

### Protective effect of lcCCL28-25 on neutropenic mouse thigh S. aureus infection model.

**FIG 7:**
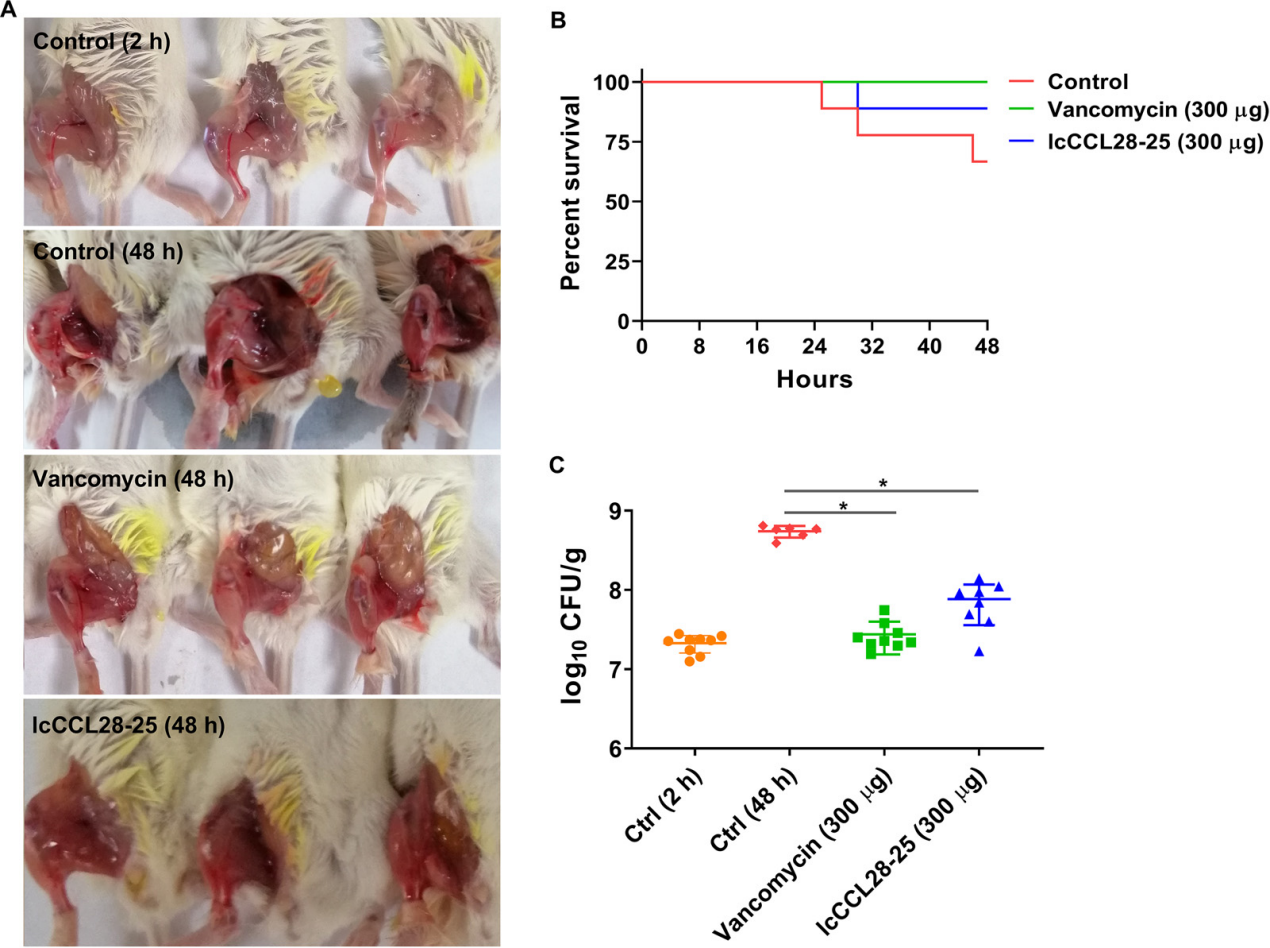
Neutropenic mouse thigh infection model. (A) Infection status of S. aureus USA300 in mouse thigh. (B) Survival plot of mice in different treatment groups. (C) Colony counts after administration of PBS, vancomycin, or lcCCL28-25 in neutropenic mouse thigh infection model. Each data point represents the value for a single mouse. All the experiments were performed in triplicate. Data are presented as mean values ± standard deviations. Data were statistically analyzed via unpaired nonparametric Mann-Whitney *U* test; *, *P < *0.001.

The *in vivo* effect of lcCCL28-25 on methicillin-resistant S. aureus standard strain USA300 was investigated using a neutropenic mouse thigh infection model. At 4 h postinfection, the mice were lethargic, rarely eating or drinking, and their hind thighs were swollen, with dragging of the thigh when walking. Three mice in the control group (phosphate-buffered saline [PBS]), one mouse in the lcCCL28-25 group, and none of the mice in the vancomycin group died after 48 h of infection (Fig. 7B). At the infection termination point, representative samples from each group were randomly selected, and the results are shown in Fig. 7A. At 48 h postinfection, there was significant redness and congestion in the thigh compared to 2 h postinfection. These symptoms were significantly alleviated in the vancomycin and lcCCL28-25 intraperitoneal-injection groups. The effects of vancomycin and lcCCL28-25 against S. aureus USA300 in the thigh infection model are shown in Fig. 7C. There was an 0.86 log_10_ CFU/g reduction in the average bacterial load in the thighs of lcCCL28-25-treated mice compared to the bacterial load in the control group, and a 1.30 log_10_ CFU/g reduction in the vancomycin group.

### Antibacterial mechanism of lcCCL28-25.

**FIG 8:**
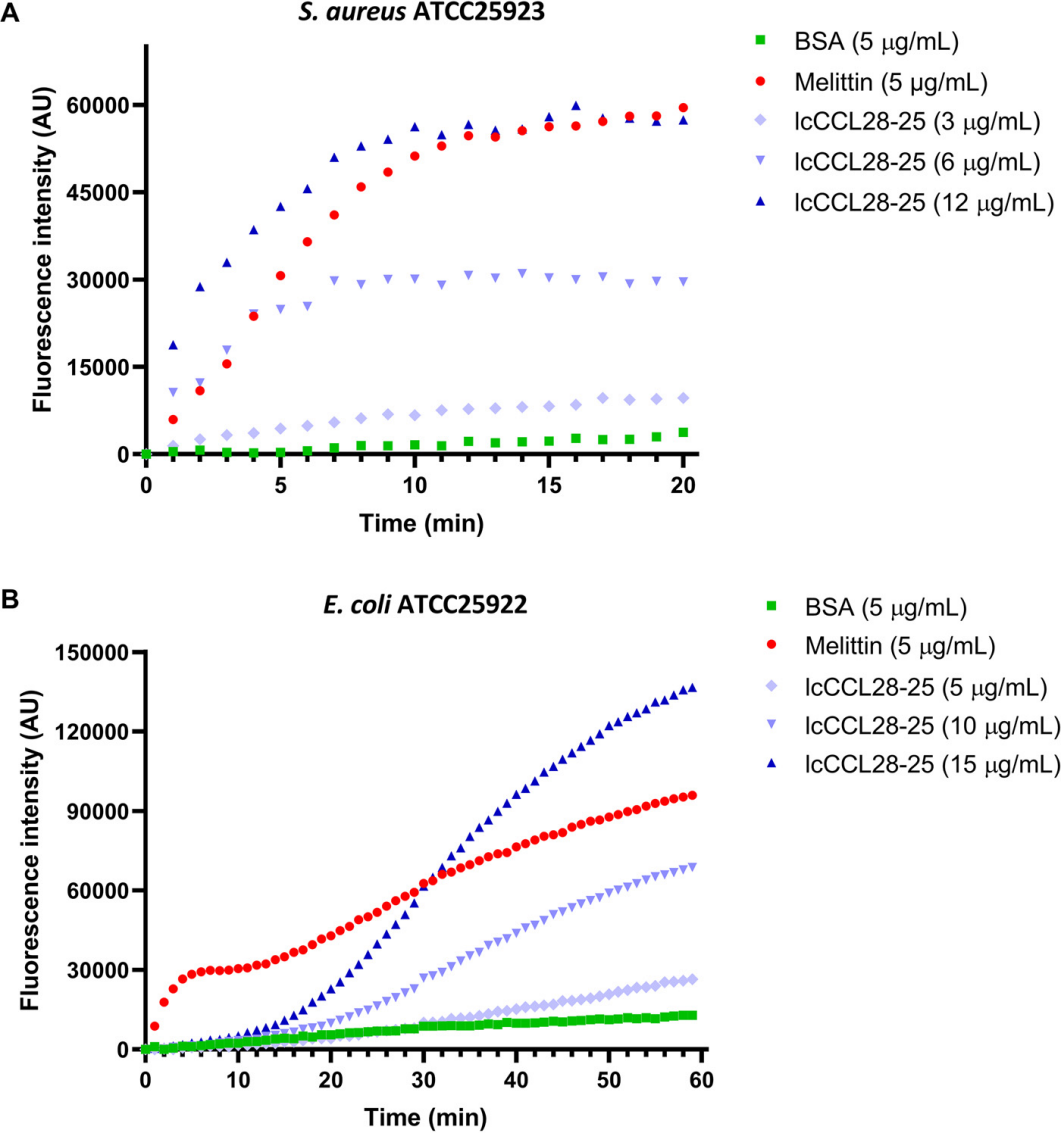
LcCCL28-25 disrupts bacterial cell membrane permeability. (A) S. aureus ATCC 25923. (B) E. coli ATCC 25922. Bacterial ingestion of SYTOX green was quantified by measuring intracellular fluorescence every 1 min. AU, absorbance units.

To determine the antibacterial mechanism of lcCCL28-25, the effects of lcCCL28-25 on the permeability of bacterial membranes were investigated using the SYTOX green method. SYTOX green, a dye that fluoresces when it binds to nucleic acids, can enter cells only through damaged cell membranes. As shown by the results in Fig. 8A, the fluorescence intensity of S. aureus increased rapidly within 20 min of lcCCL28-25 treatment, consistent with the effect of melittin. Additionally, S. aureus cells treated with the other six peptides exhibited similar fluorescence trends (Fig. S16). These findings suggested that lcCCL28-25 destroyed the S. aureus cell membrane.

However, the fluorescence intensity of E. coli cells did not increase significantly in a short time (20 min) following lcCCL28-25 treatment, in contrast to the results for melittin (Fig. 8B). The fluorescence intensity of E. coli gradually increased during coincubation (Fig. 8B), indicating that lcCCL28-25 damaged the E. coli membrane; however, the damage occurred more slowly than in S. aureus, or it may not have been direct membrane damage. Additionally, E. coli cells treated with omCK11-31, drCCL27b-24, and chCCL27a-26 exhibited fluorescence trends similar to the trends in E. coli cells treated with lcCCL28-25 (Fig. S17A, C, and F), suggesting that these peptides may have more similar antibacterial mechanisms. Notably, the fluorescence intensity of E. coli cells increased rapidly within 20 min of treatment with omCCL28-like-23, smCCL27b-25, or trCCL28-29, consistent with the results for melittin (Fig. S17B, D, and E). This finding suggested that omCCL28-like-23, smCCL27b-25, and trCCL28-29 may damage cell membranes rapidly and directly.

**FIG 9:**
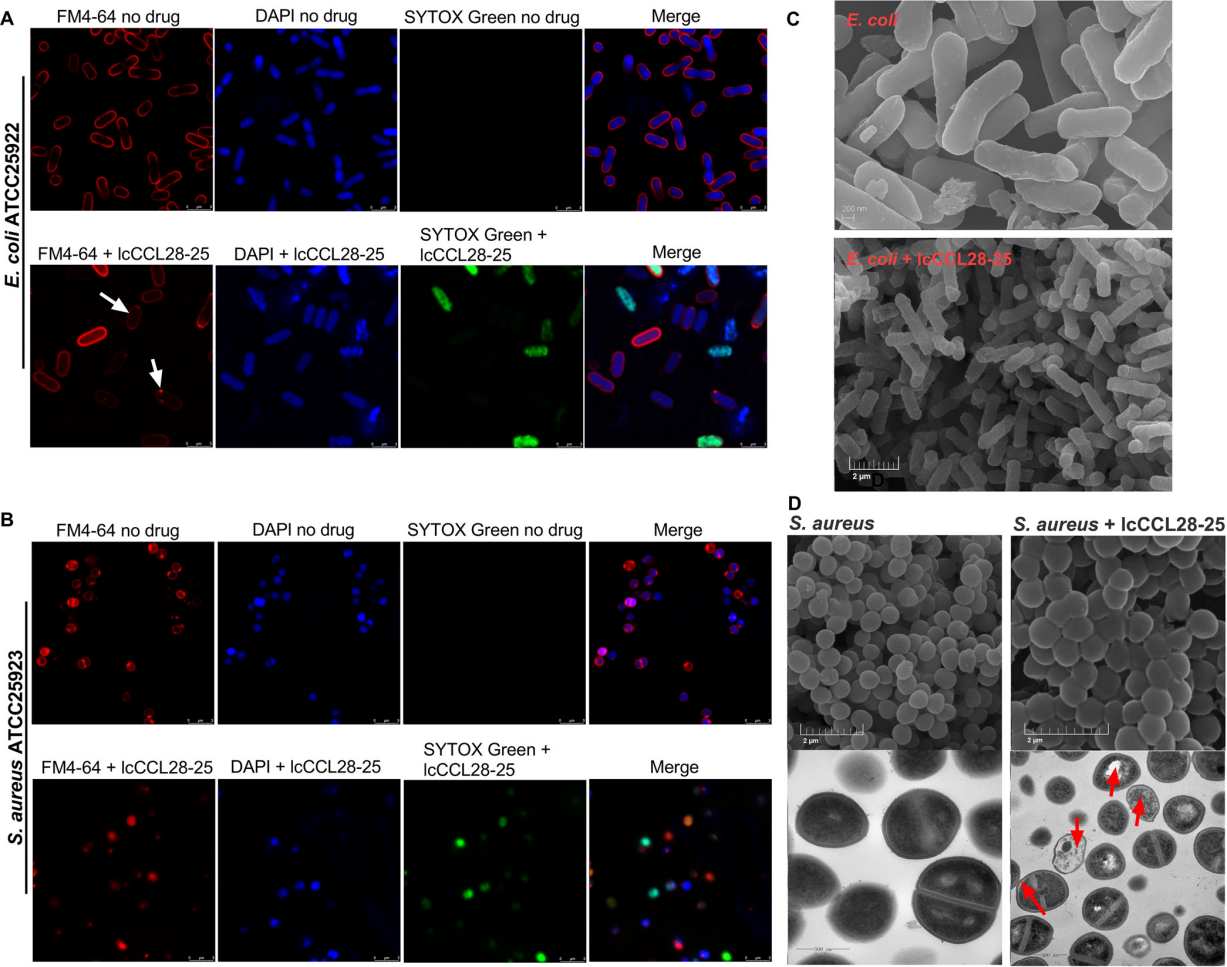
Fluorescence and electron microscopy. (A, B) Fluorescence microscopy of E. coli ATCC 25922 cells (A) and S. aureus ATCC 25923 cells (B) grown without treatment or with lcCCL28-25 and stained with FM4-64, DAPI, and SYTOX green. White arrows indicate membrane-related fluorescence spots. (C, D) SEM of E. coli ATCC 25922 cells (C) and SEM and TEM of S. aureus ATCC 25923 cells (D) that were untreated or treated with lcCCL28-25. Red arrows indicate membrane deformation and detachment and also the bright areas in the cytoplasm. The electron microscopy scans were completed in duplicate, and one typical result is shown.

Superresolution stimulated emission depletion (STED) fluorescence microscopy was used to observe permeabilized cells of E. coli ATCC 25922 and S. aureus ATCC 25923 at 15 min after treatment with lcCCL28-25 using the membrane dye FM4-64 and nucleic acid stains DAPI (4′,6-diamidino-2-phenylindole) and SYTOX green. The nucleoids stained with DAPI were significantly unaffected by lcCCL28-25 (Fig. 9A and B), but many treated cells showed strong green fluorescence in the presence of SYTOX green (Fig. 9A and B), demonstrating permeability effects on the inner and outer cell membranes. When stained with FM4-64, some of the treated cells exhibited membrane-related red fluorescence spots and weaker membrane staining (Fig. 9A, white arrows), indicating that the membrane structure had been disrupted.

Furthermore, after 15 min of treatment with 5 μg/mL lcCCL28-25, the majority of E. coli cells showed obvious membrane depressions, while a few exhibited holes (Fig. 9C). Although scanning electron microscopy (SEM) revealed no significant damage to the membrane surface of S. aureus cells after 15 min of treatment with 5 μg/mL lcCCL28-25, the cells exhibited some morphologic changes and accumulated more extracellular matrix (Fig. 9D), which could be due to intracellular content leakages. Transmission electron microscopy (TEM) revealed significant alterations in membrane structure, including membrane deformation and detachment, as well as bright areas in the cytoplasm (Fig. 9D, red arrows).

## DISCUSSION

CC chemokines are a large subfamily of chemokines that play an important role in the innate immune system. However, progress in studying CC subgroup chemokines in teleosts is limited due to their variability and low molecular weight (29). Additionally, the functions of these putative chemokines also remain ambiguous. Currently, only one CC chemokine, O. mykiss omCK11, has been identified as a CCL27/28 chemokine. It has been demonstrated to have strong antimicrobial activity against a variety of pathogens, rather than chemotaxis toward unstimulated leukocyte populations from central immune organs or mucosal tissues (27), which opens a new chapter in the study of antimicrobial chemokines in fish. Notably, while both human and mouse CCL28 showed broad-spectrum bactericidal activity against bacteria and fungi, CCL27 did not, killing only Candida albicans at high concentrations (30). In contrast to those of mammals, fish CCL27 proteins examined in this study, including D. rerio CCL27b, S. maximus CCL27b, and C. harengus CCL27a, possessed antibacterial domains, albeit with little activity (Table 1). Although the exact number of groups with direct evolutionary homology between fish and mammalian chemokines is currently unknown, a large number of chemokines from bony fishes have been identified, including many specific CC chemokines. These findings suggest that teleost fish chemokines may represent a vast antibacterial protein library worth further exploration.

The molecular weights of omCK11 and other selected proteins in this study were between 8.20 and 9.09 kDa, which is higher than the molecular weights of the majority of AMPs. Obtaining an antibacterial domain is critical for reducing the immunogenicity of antibacterial proteins and increasing their druggability. Chemokines have a flexible N terminus and N-terminal loop, a three-stranded antiparallel β-sheet, and an α-helix C-terminal (31). The N terminus of chemokines, which determines the binding and activation of receptors, is a key structural basis for the formation of chemotactic gradients required for targeted cell migration (31, 32). Therefore, which domain might exhibit antibacterial activity? It was reported that the C-terminal α-helix region (amino acids 51 to 70) of human CCL20 exhibited significant antibacterial activity due to its high cationic and amphiphilic properties (15). AMPs have a lower likelihood of inducing drug resistance due to their preferential attack on the cell membrane (33). Extensive structure-activity studies have revealed that the net charge, hydrophobicity, and amphiphilicity are the most critical physicochemical and structural factors determining the antibacterial activity and cell selectivity (34). Although the C-terminal similarities of CCL27/28 proteins in different fish species were extremely low (Fig. 2A), it was surprising that they were both cationic and amphipathic (Fig. 1B and C and Fig. 2C, F, I, L, O, and R), with almost all of them exhibiting an α-helical structure in the bacterial membrane-mimicking environment but retaining a random coil structure in a water solution (Fig. 1D and Fig. 2D, G, J, M, P, and S). These findings suggested their potential antibacterial activity.

Antibacterial activity against Gram-negative and Gram-positive bacteria was demonstrated by the synthetic C-terminal peptides in the antibacterial assay (Table 1). Surprisingly, the antibacterial activity of lcCCL28-25, derived from L. crocea, was the strongest (Table 1). Perhaps this is due to its unique amino acid and structural composition and other factors that warrant further investigation. Additionally, its druggability was enhanced by its practically insignificant level of hemolysis (Fig. 4) and extremely low cytotoxicity (Fig. 5). Additionally, it was noted that lcCCL28-25 exhibited better antibiofilm formation and clearance activities on P. aeruginosa FRD1 than the other six peptides under the same sub-MIC conditions (Fig. 6 and Fig. S15), suggesting that it might be used as a more effective biofilm removal agent.

AMPs exert antibacterial effects primarily by physically disrupting membranes, dissipating electrochemical potential, inducing lipid asymmetry, and destroying important metabolites and cell components, resulting in cell atrophy and death (35). AMPs that primarily target membrane damage, such as melittin, exhibit rapid bactericidal kinetics, whereas some AMPs and antibiotics that primarily target intracellular components exhibit slow killing kinetics (36, 37). Bactericidal kinetics studies conducted *in vitro* revealed that lcCCL28-25 exhibited rapid bactericidal kinetics against Gram-positive bacteria and was capable of reducing CFU counts by more than half within 10 min (Fig. 3A). At a concentration of 4× MIC, its ability to increase the membrane permeability of S. aureus ATCC 25923 was comparable to that of melittin (5 μg/mL) (Fig. 8A). Additionally, using fluorescence microscopy and electron microscopy (Fig. 9B and D), S. aureus cells treated with 5 μg/mL lcCCL28-25 for 15 min were seen to have significant changes in membrane structure, which could result in intracellular content leakage (Fig. 9D). SYTOX green assay also found that the other six peptides exhibited rapid membrane disruption that was consistent with the effects of melittin, despite differences in their antibacterial activities (Fig. S16). In summary, the antibacterial mechanism of the seven peptides was typical membrane disruption and permeation. In comparison, lcCCL28-25 showed relatively slow killing kinetics against Gram-negative bacteria, with 50% killing achieved after more than 1 h at the 4× MICs (Fig. 3B). Even at a concentration of 15 μg/mL, it failed to significantly increase the SYTOX green signal in E. coli cells within 15 min, in contrast to the results for melittin (Fig. 8B). However, after 15 min of treatment with 5 μg/mL lcCCL28-25, the membrane structure of E. coli cells was disrupted, showing obvious depressions and holes (Fig. 9C), membrane-associated red fluorescence spots, and weaker membrane staining (Fig. 9A). Therefore, lcCCL28-25 disrupted the membrane of Gram-negative bacteria, but whether this damage was a decisive target or a subsequent chain reaction requires additional investigation. Unlike lcCCL28-25, omCCL28-like-23, smCCL27b-25, and trCCL28-29 treatment rapidly increased the SYTOX green fluorescence signals of cells within a short period of time (consistent with the results for melittin) (Fig. S17B, D, and E). These peptides are more likely to exert their antimicrobial activity through membrane permeation and disruption, and thus, resistance is less likely to occur.

Indeed, a large proportion of reported AMPs did not exhibit effective antimicrobial activity *in vivo* due to the complexity of the animal environment and the stability of the peptides in the presence of physiological salts and serum (6, 38, 39). High concentrations of salt ions can weaken the adsorption of AMPs to bacterial cell membranes, thereby reducing their bactericidal ability. Although the activity of lcCCL28-25 against S. aureus USA300 was affected by Na^+^ and Ca^2+^, it still maintained an acceptable activity in the presence of the two ions (Table 2). Studies have shown that Na^+^ and Ca^2+^ can not only hinder the electrostatic interaction between AMPs and bacterial membranes but also compete with peptides for the binding site of lipopolysaccharide (LPS) (40, 41), which may be a crucial reason why the activity of lcCCL28-25 is inhibited by these two ions. Additionally, mouse serum, as a mixture of various salts, tends to have a significant inhibitory effect on antibacterial activity. As expected, the activity of lcCCL28-25 was significantly inhibited by serum. However, the stability of lcCCL28-25 exposed to serum was comparable to that of melittin, retaining acceptable activity in 20% serum (Table 2).

In this study, we also demonstrated that lcCCL28-25 had a potent protective effect in thighs of neutropenic mice infected with S. aureus, though it was less effective than vancomycin (Fig. 7). Although lcCCL28-25 was not completely inactivated in the presence of serum (Table 2), it remains to be determined whether the effectiveness of lcCCL28-25 depends on its antibacterial activity or immune stimulation in the complex *in vivo* environment. These findings provide a basis for the preclinical study of lcCCL28-25.

In summary, this study performed structure-function comparative analyses of the O. mykiss antibacterial chemokine CK11 that was previously described. Different from the primary function of a chemokine, the second identity of CCL28 is that of a direct antibacterial protein, indicating its critical function in the innate immunity of L. crocea. Its C-terminal segment, named lcCCL28-25, exhibited acceptable broad-spectrum antibacterial activity, antibiofilm activity, limited hemolytic activity, and low cytotoxicity *in vitro*. It also conferred a protective effect on the thighs of neutropenic mice infected with S. aureus. lcCCL28-25 destroyed bacteria by disrupting the integrity and membrane permeability of the cell membrane.

## MATERIALS AND METHODS

### Preparation of bacterial strains, blood sample, and animals.

P. aeruginosa PAO1 and P. aeruginosa clinical strain FRD1 were cultured in our laboratory. E. coli ATCC 25922, A. hydrophila ATCC 7966, S. aureus ATCC 25923, K. pneumoniae CMCC46117, S. agalactiae ATCC 13813, S. pneumoniae ATCC 49619, and methicillin-resistant S. aureus ATCC 43300 were purchased from the American Type Culture Collection (ATCC). The methicillin-resistant S. aureus standard strain USA300 was obtained from Wenyu Han (College of Veterinary Medicine, Jilin University, China).

A fresh human blood sample was obtained from the Hospital of Ocean University of China (no. D023302).

Eight-week-old female BALB/c mice weighing 16 to 19 g, without specific pathogens, were purchased from Vital River Laboratory Animal Technology Co., Ltd. (Beijing, China). Animal experiments were performed in line with the international moral principles and guidelines for institutional animal care and use of laboratory animals (42). All animal procedures conformed to the guidelines of the Animal Ethics Committee of the School of Medicine and Pharmacy, Ocean University of China (OUC-SMP-2021-05-01).

### Preparation of peptides.

All peptides used in this study were synthesized by the GenScript Biotech Corporation (Nanjing, China).

### Three-dimensional model and charge analysis of protein.

The protein structure was evaluated through homology modeling using SWISS-MODEL (https://swissmodel.expasy.org/). The structure with PDB identifier (ID) 7JH1 was used as the template for predicting the three-dimensional models of omCK11, drCCL27b, and lcCCL28, whereas the structure with PDB ID 6CWS was used as the template for omCCL28-like and smCCL27b and the structures with PDB IDs 2KUM and 1DOM were used as the templates for trCCL28 and chCCL27a, respectively. The PDB files obtained and the net charge distributions of proteins were described by utilizing PyMOL. The net charges of peptides were analyzed in the online software NovoPro (https://www.novopro.cn/tools/protein_iep.html). The helical wheel analysis was performed by using the online software heliQuest (https://heliquest.ipmc.cnrs.fr/cgi-bin/ComputParams.py).

### CD spectroscopy.

The circular dichroism (CD) spectroscopic analysis of peptides at 37°C in the far-UV range of 190 to 240 nm was performed using a Jasco J-1500 spectropolarimeter (Jasco, Japan). The concentration of peptides was 200 μg/mL, and the light diameter of the quartz cuvette was 10 mm. The secondary structures of peptides were measured in water and in 50% (vol/vol) TFE solution. Measurements were performed in triplicate for each sample.

### MIC assays.

The MIC assays were performed as previously described (43). Briefly, peptides were plated in a 2-fold dilution series (50 μL/well) of Mueller-Hinton broth (MHB) in Costar 96-well cell culture plates (Corning, NY, USA). Three repeats were set for each concentration. E. coli ATCC 25922, A. hydrophila ATCC 7966, P. aeruginosa PAO1, P. aeruginosa FRD1, S. aureus ATCC 25923, S. agalactiae ATCC 13813, S. pneumoniae ATCC 49619, methicillin-resistant S. aureus ATCC 43300, and methicillin-resistant S. aureus USA300 were cultured in Luria-Bertani (LB) medium, while K. pneumoniae CMCC46117 was cultured in tryptic soy broth (TSB) medium. Incubation was done at 37°C and 150 × *g* for 12 h. Bacteria were transferred to fresh MHB at a ratio of 1% (vol/vol) and incubated to reach a logarithmic growth phase. Then, 50 μL of a bacterial suspension at 4 × 10^6^ CFU/mL was added to each well. After incubation at 37°C for 22 ± 2 h, turbidity was measured using an EnSpire multimode plate reader (PerkinElmer, Singapore) at 595 nm. MIC values were taken as the lowest peptide concentrations at which visible bacterial growths were completely inhibited.

To evaluate the effects of salts and mouse serum on the antimicrobial activity of lcCCL28-25 against S. aureus USA300, MIC values were also determined in the presence of different salts at physiological concentrations (150 mM NaCl, 4.5 mM KCl, 6 μM NH_4_Cl, 1 mM MgCl_2_, or 2.5 mM CaCl_2_) or mouse serum (1%, 10%, and 20% [vol/vol]), using the same method described above.

### Kill-curve kinetics.

Consistent with previous reports, time-kill curves of lcCCL28-25 against Gram-negative and Gram-positive bacteria were performed under 4× MIC conditions (44). Bacteria were incubated to the logarithmic growth stage. Bacterial cells in the logarithmic growth phase were diluted to achieve log(CFU/mL) values of ~5 to 6 and incubated with lcCCL28-25 for 1, 2, 4, 8, 16, 30, 60, 120, and 240 min. At each time point, 50-μL amounts of the samples were diluted to different concentrations and plated onto agar plates. After overnight incubation, the numbers of colonies on the plates were counted.

### Hemolytic activities of peptides against hRBCs.

Hemolytic activities were determined as previously described (45). Briefly, fresh human red blood cells (hRBCs) were washed thrice using PBS. Peptides were gradient diluted to 2-fold final concentrations using PBS and added to a 96-well plate at 100 μL/well. An equal volume of cell suspension (4% [wt/vol] in PBS) was added to each well, resulting in final peptide concentrations of 6.25, 12.5, 25, 50, 100, 250, and 500 μg/mL. After incubation at 37°C for 1 h, the supernatant was obtained by centrifugation at 1,000 × *g* for 4 min, and its absorbance measured at 540 nm. The value for 100% hemolysis was established using 0.1% (vol/vol) Triton X-100, while the value for 0% was determined using PBS.

### Analysis of cytotoxicity of lcCCL28-25 to mammalian cells.

The MTT [3-(4,5-dimethyl-2-thiazolyl)-2,5-diphenyl-2H-tetrazolium bromide] assay was performed to assess the cytotoxic effects of lcCCL28-25 on RAW264.7 and HEK293T cells. Cells were precultured in Dulbecco modified Eagle medium (DMEM) supplemented with 10% fetal bovine serum (FBS) and incubated at 37°C in a 5% CO_2_ atmosphere. For this test, cells were seeded in 96-well plates at 10^4^ cells/well and incubated for 24 h. The same volume of lcCCL28-25 or melittin with different concentrations was added to each well to final concentrations of 1.56, 3.125, 6.25, 12.5, 25, 50, 100, and 200 μg/mL. After incubation for 1 h at 37°C in a 5% CO_2_ atmosphere, the medium was replaced with a fresh medium containing 10% (vol/vol) MTT solution (5 mg/mL). After incubation for 4 h, the formed crystals were dissolved in dimethyl sulfoxide (DMSO) and the absorbance measured at 550 nm.

### Biofilm inhibition and eradication activities.

*In vitro* static biofilm models have been reported in many studies (45–47). A Pseudomonas aeruginosa mucoid strain, FRD1, isolated from a cystic fibrosis patient, was used for the biofilm inhibition and eradication assay. The FRD1 strain was shock cultured to logarithmic growth stage at 37°C and 150 × *g*. After cleaning using PBS and centrifuging thrice, the bacteria were resuspended in MHB medium (with 0.2% glucose) and gradient diluted to an OD_600_ value of ~0.01.

For the biofilm inhibition assay, 90-μL amounts of bacterial suspensions and 10-μL amounts of increasing concentrations of the peptide were added to a 96-well plate and coincubated at 37°C for 24 h. Then, bacterial biofilms were quantified as follows: (i) planktonic bacteria were removed by washing thrice using PBS, (ii) biofilm bacteria were fixed in 100 μL of 4% paraformaldehyde at room temperature for 15 min and (iii) stained with 100 μL of 0.1% crystal violet for 30 min, (iv) the dye was removed, followed by washing twice using PBS, adding 200 μL of 95% ethanol to each well to dissolve the crystal violet, and finally, (v) absorbance was measured at 590 nm using an EnSpire multimode plate reader (PerkinElmer, Singapore).

For the eradication assay, biofilms of the FRD1 strain were grown in a 96-well plate. Briefly, 100-μL amounts of the bacterial suspension were plated and incubated at 37°C for 24 h. Then, wells containing the biofilms were washed thrice using PBS. The peptide was serially diluted in MHB medium (with 0.2% glucose) and added to the 96-well plates (100 μL/well). The plates were incubated for 2 h at 37°C. Finally, bacterial biofilms were quantified as described above for the biofilm inhibition assay.

### Neutropenic mouse thigh infection model.

Female BALB/c mice were used to construct a neutropenic murine thigh infection model as previously published (48–50). Briefly, mice (*n* = 9/group) were rendered neutropenic by consecutive intraperitoneal injections of 150 and 100 mg/kg of body weight cyclophosphamide at 4 and 1 day before bacterial injection (51). An S. aureus USA300 inoculum (~1.5 × 10^6^ CFU/mL) was prepared by diluting logarithmic phase bacteria with fresh MHB. Each mouse was infected by intramuscular injection of 0.1 mL of the inoculum (~1.5 × 10^5^ CFU) into the right thigh muscles. At 2 h postinfection, 0.1 mL of lcCCL28-25 peptide (300 μg) or vancomycin hydrochloride (300 μg, 15 mg/kg) was administered by intraperitoneal injection. The control group was injected with an equal volume of PBS. Untreated control mice were sacrificed at the start of treatment to establish the baseline bacterial burden. Mice in the three treatment groups (PBS, vancomycin, and lcCCL28-25) were sacrificed at 48 h postinfection. Their right thighs were aseptically harvested, placed in sterile saline and homogenized using a Bioprep-6 homogenizer (Allsheng, Hangzhou, China). After being serially diluted to 1:10 in sterile saline, 100-μL amounts of tissue suspensions at various concentrations were plated on LB agar to determine bacterial counts after incubation at 37°C for 24 h.

### Bacterial cell membrane permeability assay.

The SYTOX green staining assay was performed to assess peptide-associated membrane damage (47, 52). E. coli ATCC 25922 and S. aureus ATCC 25923 cells in the mid-logarithmic phase were collected, washed thrice, and resuspended (5 × 10^7^ CFU/mL) in buffer B (10 mM Tris-HCl, pH 7.4, at 25°C). The bacterial suspensions were mixed with 5 μM SYTOX green and incubated for 15 min at room temperature in the dark. Then, the mixtures were added to black, clear-bottom 96-well plates at a density of 100 μL/well. After peptide addition, SYTOX green uptake was monitored using an EnSpire multimode plate reader (PerkinElmer, Singapore) with an excitation wavelength of 485 nm and an emission wavelength of 520 nm. Each measurement interval was 1 min, totaling 30 to 60 times. Melittin was used as the positive control, while bovine serum albumin (BSA) was the negative control.

### Fluorescence microscopy.

E. coli ATCC 25922 and S. aureus ATCC 25923 cells were grown overnight in LB medium and subcultured at 37°C and 150 × *g* to achieve the mid-logarithmic phase (OD_600_ was ~0.4 to 0.6). After washing thrice using buffer B (10 mM Tris-HCl, pH 7.4, at 25°C), lcCCL28-25 (5 μg/mL) was added to the bacterial suspensions and coincubated at room temperature for 15 min. Then, the fluorescent dye and stains (FM4-64 [1 μg/mL], SYTOX green [0.5 μM], and DAPI [2 μg/mL]) were added, after which the broth was incubated at 0°C for 10 to 15 min (DAPI and SYTOX green) or 45 to 60 min (FM4-64). Cells were obtained by centrifugation, washed, and resuspended in PBS (20 μL). Subsequently, 1 μL of the bacterial suspension was dropped onto a precleaned slide (1 mm thick) and covered with a cover glass (0.17 mm thick). Then, samples were imaged by a Leica TCS SP8 STED microscope, with laser excitation for DAPI (405 nm), SYTOX green (514 nm), or FM4-64 (520 nm) and a photomultiplier or hybrid detector. A STED laser was applied at 660 nm for SYTOX green and 775 nm for FM4-64.

### Electron microscopy.

E. coli ATCC 25922 and S. aureus ATCC 25923 cells were cultured and coincubated with or without lcCCL28-25 as described above for fluorescence microscopy.

For scanning electron microscopy (SEM), bacterial cells were fixed with 2.5% glutaraldehyde in PBS (pH 7.35) and attached to 12-mm cover glasses by centrifugation at 75 × *g* for 6 min using a Cytospin 2 centrifuge (Thermo Fisher Scientific, Schwerte, Germany). Then, cover glasses with attached bacteria were postfixed in 1% aqueous OsO_4_ for 30 min, dehydrated in a sequence of increasing ethanol concentrations (70%, 80%, and 100%), and critical point dried in a CPD030 (Leica Microsystems, Vienna, Austria). Next, dried samples were mounted on aluminum stubs using conductive carbon tabs, coated with 4 nm of platinum in a CCU-010 sputtering device (Safematic, Bad Ragaz, Switzerland), and imaged by a Supra 50 VP SEM at an acceleration voltage of 2 kV using the in-lens secondary electron detector (Zeiss, Oberkochen, Germany) (53).

For transmission electron microscopy (TEM), bacterial cells were obtained by centrifugation after overnight fixation in 2.5% glutaraldehyde (pH 7.35, in PBS) at 4°C. Cells from the pellet were drawn into cellulose capillary tubes, immersed in 1-hexadecene, sliced into tubes of about 4 mm in length, transferred into 6-mm aluminum specimen carriers with an indentation of 5 mm by 0.15 mm, covered with a flat 6-mm aluminum specimen carrier dipped in 1-hexadecene, and frozen with an EM HPM100 high-pressure freezing machine (Leica Microsystems, Vienna, Austria). Freeze substitution (EM AFS2 freeze-substitution unit; Leica Microsystems) was performed in anhydrous acetone containing 1% OsO_4_ at −90°C for 9 h, −60°C for 6 h, −30°C for 3 h, and 0°C for 1 h with temperature transition gradients of 30°C per hour. After washing thrice using anhydrous acetone, samples were incubated in 1% uranyl-acetate (from 20% stock solution in anhydrous methanol) for 1 h at 0°C. Then, samples were rinsed thrice in anhydrous acetone and embedded overnight in 66% Epon-Araldite (Sigma-Aldrich, Buchs, Switzerland) in anhydrous acetone at 4°C, 100% Epon-Araldite at room temperature for 1 h, and polymerized at 60°C for at least 28 h. Thin sections were poststained with Reynolds lead citrate and imaged by a CM100 or Tecnai G2 spirit TEM (Thermo Fisher Scientific, Eindhoven, The Netherlands) at 80 or 120 kV acceleration voltage, respectively. Imaging was performed using a side-mounted Orius 1000 charge-coupled-device (CCD) camera (4,000 by 2,600 pixels; Gatan, Munich, Germany) (53).

### Statistical analysis.

Statistical analyses for data obtained from *in vitro* and *in vivo* experiments were performed using the two-tailed unpaired Student *t* test and unpaired nonparametric Mann-Whitney *U* test, respectively. A *P* value of <0.05 was considered significant. Group sizes, reproducibility, and *P* values for each experiment are given in the figure legends.

## References

[1] Nieuwlaat R, Mbuagbaw L, Mertz D, Burrows LL, Bowdish DME, Moja L, Wright GD, Schünemann HJ. 2021. Coronavirus disease 2019 and antimicrobial resistance: parallel and interacting health emergencies. Clin Infect Dis 72:1657–1659. doi:10.1093/cid/ciaa773. 32544232 PMC7337675

[2] El-Sayed Ahmed MAE-G, Zhong L-L, Shen C, Yang Y, Doi Y, Tian G-B. 2020. Colistin and its role in the Era of antibiotic resistance: an extended review (2000–2019). Emerg Microbes Infect 9:868–885. doi:10.1080/22221751.2020.1754133. 32284036 PMC7241451

[3] Taguchi T, Mukai K. 2019. Innate immunity signalling and membrane trafficking. Curr Opin Cell Biol 59:1–7. doi:10.1016/j.ceb.2019.02.002. 30875551

[4] Sun L, Wang X, Saredy J, Yuan Z, Yang X, Wang H. 2020. Innate-adaptive immunity interplay and redox regulation in immune response. Redox Biol 37:101759. doi:10.1016/j.redox.2020.101759. 33086106 PMC7575795

[5] Mookherjee N, Anderson MA, Haagsman HP, Davidson DJ. 2020. Antimicrobial host defence peptides: functions and clinical potential. Nat Rev Drug Discov 19:311–332. doi:10.1038/s41573-019-0058-8. 32107480

[6] Thapa RK, Diep DB, Tønnesen HH. 2020. Topical antimicrobial peptide formulations for wound healing: current developments and future prospects. Acta Biomater 103:52–67. doi:10.1016/j.actbio.2019.12.025. 31874224

[7] Xiao X, Zhu W, Zhang Y, Liao Z, Wu C, Yang C, Zhang Y, Xiao S, Su J. 2021. Broad-spectrum robust direct bactericidal activity of fish IFNφ1 reveals an antimicrobial peptide-like function for type I IFNs in vertebrates. J Immunol 206:1337–1347. doi:10.4049/jimmunol.2000680. 33568398

[8] Yin Q, Wu S, Wu L, Wang Z, Mu Y, Zhang R, Dong C, Zhou B, Zhao B, Zheng J, Sun Y, Cheng X, Yang L. 2020. A novel in silico antimicrobial peptide DP7 combats MDR Pseudomonas aeruginosa and related biofilm infections. J Antimicrob Chemother 75:3248–3259. doi:10.1093/jac/dkaa308. 32737484

[9] Hughes CE, Nibbs RJB. 2018. A guide to chemokines and their receptors. FEBS J 285:2944–2971. doi:10.1111/febs.14466. 29637711 PMC6120486

[10] Legler DF, Thelen M. 2016. Chemokines: chemistry, biochemistry and biological function. Chimia (Aarau) 70:856–859. doi:10.2533/chimia.2016.856. 28661356

[11] Yount NY, Waring AJ, Gank KD, Welch WH, Kupferwasser D, Yeaman MR. 2007. Structural correlates of antimicrobial efficacy in IL-8 and related human kinocidins. Biochim Biophys Acta 1768:598–608. doi:10.1016/j.bbamem.2006.11.011. 17208195

[12] Yeaman MR, Tang YQ, Shen AJ, Bayer AS, Selsted ME. 1997. Purification and *in vitro* activities of rabbit platelet microbicidal proteins. Infect Immun 65:1023–1031. doi:10.1128/IAI.65.3.1023-1031.1997. 9038312 PMC175084

[13] Tang YQ, Yeaman MR, Selsted ME. 2002. Antimicrobial peptides from human platelets. Infect Immun 70:6524–6533. doi:10.1128/IAI.70.12.6524-6533.2002. 12438321 PMC132966

[14] Collin M, Linge HM, Bjartell A, Giwercman A, Malm J, Egesten A. 2008. Constitutive expression of the antibacterial CXC chemokine GCP-2/CXCL6 by epithelial cells of the male reproductive tract. J Reprod Immunol 79:37–43. doi:10.1016/j.jri.2008.08.003. 18809212

[15] Nguyen LT, Chan DI, Boszhard L, Zaat SA, Vogel HJ. 2010. Structure-function studies of chemokine-derived carboxy-terminal antimicrobial peptides. Biochim Biophys Acta 1798:1062–1072. doi:10.1016/j.bbamem.2009.11.021. 20004172

[16] Egesten A, Eliasson M, Johansson HM, Olin AI, Morgelin M, Mueller A, Pease JE, Frick IM, Bjorck L. 2007. The CXC chemokine MIG/CXCL9 is important in innate immunity against *Streptococcus pyogenes*. J Infect Dis 195:684–693. doi:10.1086/510857. 17262710

[17] Dai C, Basilico P, Cremona TP, Collins P, Moser B, Benarafa C, Wolf M. 2015. CXCL14 displays antimicrobial activity against respiratory tract bacteria and contributes to clearance of *Streptococcus pneumoniae* pulmonary infection. J Immunol 194:5980–5989. doi:10.4049/jimmunol.1402634. 25964486

[18] Bird S, Tafalla C. 2015. Teleost chemokines and their receptors. Biology (Basel) 4:756–784. doi:10.3390/biology4040756. 26569324 PMC4690017

[19] Gangele K, Jamsandekar M, Mishra A, Poluri KM. 2019. Unraveling the evolutionary origin of ELR motif using fish CXC chemokine CXCL8. Fish Shellfish Immunol 93:17–27. doi:10.1016/j.fsi.2019.07.034. 31310848

[20] Castro R, Tafalla C. 2015. Overview of fish immunity, p 3–54. *In* Beck BH, Peatman E (ed), Mucosal health in aquaculture. Academic Press, London, UK.

[21] Gao A, Li L, Yan F, Lei Y, Chen J, Wu L, Ye J. 2021. Nile tilapia CXCR4, the receptor of chemokine CXCL12, is involved in host defense against bacterial infection and chemotactic activity. Dev Comp Immunol 114:103836. doi:10.1016/j.dci.2020.103836. 32835835

[22] Mu Y, Zhou S, Ding N, Ao J, Chen X. 2019. Molecular characterization of a new fish specific chemokine CXCL_F6 in large yellow croaker (*Larimichthys crocea*) and its role in inflammatory response. Fish Shellfish Immunol 84:787–794. doi:10.1016/j.fsi.2018.10.068. 30393176

[23] Pijanowski L, Verburg-van Kemenade BML, Chadzinska M. 2019. A role for CXC chemokines and their receptors in stress axis regulation of common carp. Gen Comp Endocrinol 280:194–199. doi:10.1016/j.ygcen.2019.05.004. 31075272

[24] Zhou T, Li N, Jin Y, Zeng Q, Prabowo W, Liu Y, Tian C, Bao L, Liu S, Yuan Z, Fu Q, Gao S, Gao D, Dunham R, Shubin NH, Liu Z. 2018. Chemokine C-C motif ligand 33 is a key regulator of teleost fish barbel development. Proc Natl Acad Sci USA 115:E5018–E5027. doi:10.1073/pnas.1718603115. 29760055 PMC5984497

[25] Siekmann AF, Standley C, Fogarty KE, Wolfe SA, Lawson ND. 2009. Chemokine signaling guides regional patterning of the first embryonic artery. Genes Dev 23:2272–2277. doi:10.1101/gad.1813509. 19797767 PMC2758748

[26] Bussmann J, Wolfe SA, Siekmann AF. 2011. Arterial-venous network formation during brain vascularization involves hemodynamic regulation of chemokine signaling. Development 138:1717–1726. doi:10.1242/dev.059881. 21429983 PMC3074448

[27] Muñoz-Atienza E, Aquilino C, Syahputra K, Al-Jubury A, Araújo C, Skov J, Kania PW, Hernández PE, Buchmann K, Cintas LM, Tafalla C. 2019. CK11, a teleost chemokine with a potent antimicrobial activity. J Immunol 202:857–870. doi:10.4049/jimmunol.1800568. 30610164

[28] Mishra B, Reiling S, Zarena D, Wang G. 2017. Host defense antimicrobial peptides as antibiotics: design and application strategies. Curr Opin Chem Biol 38:87–96. doi:10.1016/j.cbpa.2017.03.014. 28399505 PMC5494204

[29] Sun B, Lei Y, Cao Z, Zhou Y, Sun Y, Wu Y, Wang S, Guo W, Liu C. 2019. TroCCL4, a CC chemokine of *Trachinotus ovatus*, is involved in the antimicrobial immune response. Fish Shellfish Immunol 86:525–535. doi:10.1016/j.fsi.2018.11.080. 30521967

[30] Hieshima K, Ohtani H, Shibano M, Izawa D, Nakayama T, Kawasaki Y, Shiba F, Shiota M, Katou F, Saito T, Yoshie O. 2003. CCL28 has dual roles in mucosal immunity as a chemokine with broad-spectrum antimicrobial activity. J Immunol 170:1452–1461. doi:10.4049/jimmunol.170.3.1452. 12538707

[31] Miller MC, Mayo KH. 2017. Chemokines from a structural perspective. Int J Mol Sci 18:2088. doi:10.3390/ijms18102088. 28974038 PMC5666770

[32] Crump MP, Gong JH, Loetscher P, Rajarathnam K, Amara A, Arenzana-Seisdedos F, Virelizier JL, Baggiolini M, Sykes BD, Clark-Lewis I. 1997. Solution structure and basis for functional activity of stromal cell-derived factor-1; dissociation of CXCR4 activation from binding and inhibition of HIV-1. EMBO J 16:6996–7007. doi:10.1093/emboj/16.23.6996. 9384579 PMC1170303

[33] Bechinger B, Gorr SU. 2017. Antimicrobial peptides: mechanisms of action and resistance. J Dent Res 96:254–260. doi:10.1177/0022034516679973. 27872334 PMC5298395

[34] Wang J, Dou X, Song J, Lyu Y, Zhu X, Xu L, Li W, Shan A. 2019. Antimicrobial peptides: promising alternatives in the post feeding antibiotic era. Med Res Rev 39:831–859. doi:10.1002/med.21542. 30353555

[35] Brogden KA. 2005. Antimicrobial peptides: pore formers or metabolic inhibitors in bacteria? Nat Rev Microbiol 3:238–250. doi:10.1038/nrmicro1098. 15703760

[36] Qiao Y, Ma X, Zhang M, Zhong S. 2021. Cerocin, a novel piscidin-like antimicrobial peptide from black seabass, Centropristis striata. Fish Shellfish Immunol 110:86–90. doi:10.1016/j.fsi.2020.12.005. 33348038

[37] Wu Q, Patočka J, Kuča K. 2018. Insect antimicrobial peptides, a mini review. Toxins (Basel) 10:461. doi:10.3390/toxins10110461. 30413046 PMC6267271

[38] Ma L, Xie X, Liu H, Huang Y, Wu H, Jiang M, Xu P, Ye X, Zhou C. 2020. Potent antibacterial activity of MSI-1 derived from the magainin 2 peptide against drug-resistant bacteria. Theranostics 10:1373–1390. doi:10.7150/thno.39157. 31938070 PMC6956804

[39] Lai Z, Tan P, Zhu Y, Shao C, Shan A, Li L. 2019. Highly stabilized α-helical coiled coils kill Gram-negative bacteria by multicomplementary mechanisms under acidic condition. ACS Appl Mater Interfaces 11:22113–22128. doi:10.1021/acsami.9b04654. 31199117

[40] Zhu X, Dong N, Wang Z, Ma Z, Zhang L, Ma Q, Shan A. 2014. Design of imperfectly amphipathic α-helical antimicrobial peptides with enhanced cell selectivity. Acta Biomater 10:244–257. doi:10.1016/j.actbio.2013.08.043. 24021230

[41] Huang J, Hao D, Chen Y, Xu Y, Tan J, Huang Y, Li F, Chen Y. 2011. Inhibitory effects and mechanisms of physiological conditions on the activity of enantiomeric forms of an α-helical antibacterial peptide against bacteria. Peptides 32:1488–1495. doi:10.1016/j.peptides.2011.05.023. 21664394

[42] Couto M, Cates C. 2019. Laboratory guidelines for animal care. Methods Mol Biol 1920:407–430. doi:10.1007/978-1-4939-9009-2_25. 30737706

[43] Armas F, Di Stasi A, Mardirossian M, Romani AA, Benincasa M, Scocchi M. 2021. Effects of lipidation on a proline-rich antibacterial peptide. Int J Mol Sci 22:7959. doi:10.3390/ijms22157959. 34360723 PMC8347091

[44] Ma L, Wang Y, Wang M, Tian Y, Kang W, Liu H, Wang H, Dou J, Zhou C. 2016. Effective antimicrobial activity of Cbf-14, derived from a cathelin-like domain, against penicillin-resistant bacteria. Biomaterials 87:32–45. doi:10.1016/j.biomaterials.2016.02.011. 26897538

[45] Rajasekaran G, Dinesh Kumar S, Nam J, Jeon D, Kim Y, Lee CW, Park IS, Shin SY. 2019. Antimicrobial and anti-inflammatory activities of chemokine CXCL14-derived antimicrobial peptide and its analogs. Biochim Biophys Acta Biomembr 1861:256–267. doi:10.1016/j.bbamem.2018.06.016. 29959905

[46] Roy R, Tiwari M, Donelli G, Tiwari V. 2018. Strategies for combating bacterial biofilms: a focus on anti-biofilm agents and their mechanisms of action. Virulence 9:522–554. doi:10.1080/21505594.2017.1313372. 28362216 PMC5955472

[47] Rajasekaran G, Kim EY, Shin SY. 2017. LL-37-derived membrane-active FK-13 analogs possessing cell selectivity, anti-biofilm activity and synergy with chloramphenicol and anti-inflammatory activity. Biochim Biophys Acta Biomembr 1859:722–733. doi:10.1016/j.bbamem.2017.01.037. 28161291

[48] Liu Y, Jia Y, Yang K, Li R, Xiao X, Zhu K, Wang Z. 2020. Metformin restores tetracyclines susceptibility against multidrug resistant bacteria. Adv Sci (Weinh) 7:1902227. doi:10.1002/advs.201902227. 32596101 PMC7312304

[49] Choi S, Moon SM, Park S-J, Lee SC, Jung KH, Sung H-S, Kim M-N, Jung J, Kim MJ, Kim S-H, Lee S-O, Choi S-H, Jeong J-Y, Woo JH, Kim YS, Chong YP. 2020. Antagonistic effect of colistin on vancomycin activity against methicillin-resistant *Staphylococcus aureus* in *in vitro* and *in vivo* studies. Antimicrob Agents Chemother 64:e01925-19. doi:10.1128/AAC.01925-19. 32041713 PMC7179267

[50] Sabet M, Tarazi Z, Griffith DC. 2020. Pharmacodynamics of meropenem against *Acinetobacter baumannii* in a neutropenic mouse thigh infection model. Antimicrob Agents Chemother 64:e02388-19. doi:10.1128/AAC.02388-19. 31988101 PMC7179318

[51] Ling LL, Schneider T, Peoples AJ, Spoering AL, Engels I, Conlon BP, Mueller A, Schäberle TF, Hughes DE, Epstein S, Jones M, Lazarides L, Steadman VA, Cohen DR, Felix CR, Fetterman KA, Millett WP, Nitti AG, Zullo AM, Chen C, Lewis K. 2015. A new antibiotic kills pathogens without detectable resistance. Nature 517:455–459. doi:10.1038/nature14098. 25561178 PMC7414797

[52] Aili SR, Touchard A, Escoubas P, Padula MP, Orivel J, Dejean A, Nicholson GM. 2014. Diversity of peptide toxins from stinging ant venoms. Toxicon 92:166–178. doi:10.1016/j.toxicon.2014.10.021. 25448389

[53] Luther A, Urfer M, Zahn M, Müller M, Wang SY, Mondal M, Vitale A, Hartmann JB, Sharpe T, Monte FL, Kocherla H, Cline E, Pessi G, Rath P, Modaresi SM, Chiquet P, Stiegeler S, Verbree C, Remus T, Schmitt M, Kolopp C, Westwood MA, Desjonquères N, Brabet E, Hell S, LePoupon K, Vermeulen A, Jaisson R, Rithié V, Upert G, Lederer A, Zbinden P, Wach A, Moehle K, Zerbe K, Locher HH, Bernardini F, Dale GE, Eberl L, Wollscheid B, Hiller S, Robinson JA, Obrecht D. Chimeric peptidomimetic antibiotics against Gram-negative bacteria. Nature 576:452–458. 31645764 10.1038/s41586-019-1665-6

