# Supplementary material for: LcCCL28-25, Derived from Piscine Chemokine, Exhibits Antimicrobial Activity against Gram-Negative and Gram-Positive Bacteria In Vitro and In Vivo
Source: Microbiol Spectr. 2022 May 26;10(3):e02515-21. doi: 10.1128/spectrum.02515-21 (PMC9241943; doi:10.1128/spectrum.02515-21)
Supplement: SUPPLEMENTAL FILE 1 — Fig. S1 to S17. Download spectrum.02515-21-s001.pdf, PDF file, 0.9 MB [file spectrum.02515-21-s001.pdf]

Supplementary materials

LcCCL28-25, derived from piscine chemokine, exhibits antimicrobial activity against Gram-negative/positive bacteria *in vitro*/*in vivo*

Juanjuan Su,[a,b,c,d] Haimeng Li,[a,b,c,d] Jingyang Hu,[a,b,c,d] Danni Wang, [a,b,c,d] Fengchao Zhang,[a,b,c,d] Zheng Fu,[a,b,c,d] Feng Han[a,b,c,d#]

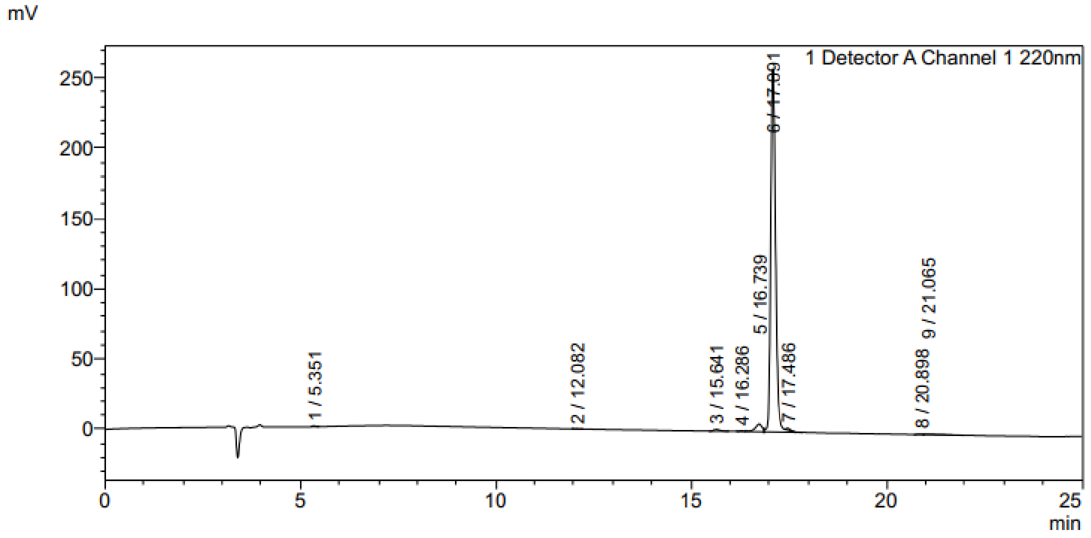

Fig. S1. Analytical HPLC for omCK11-31

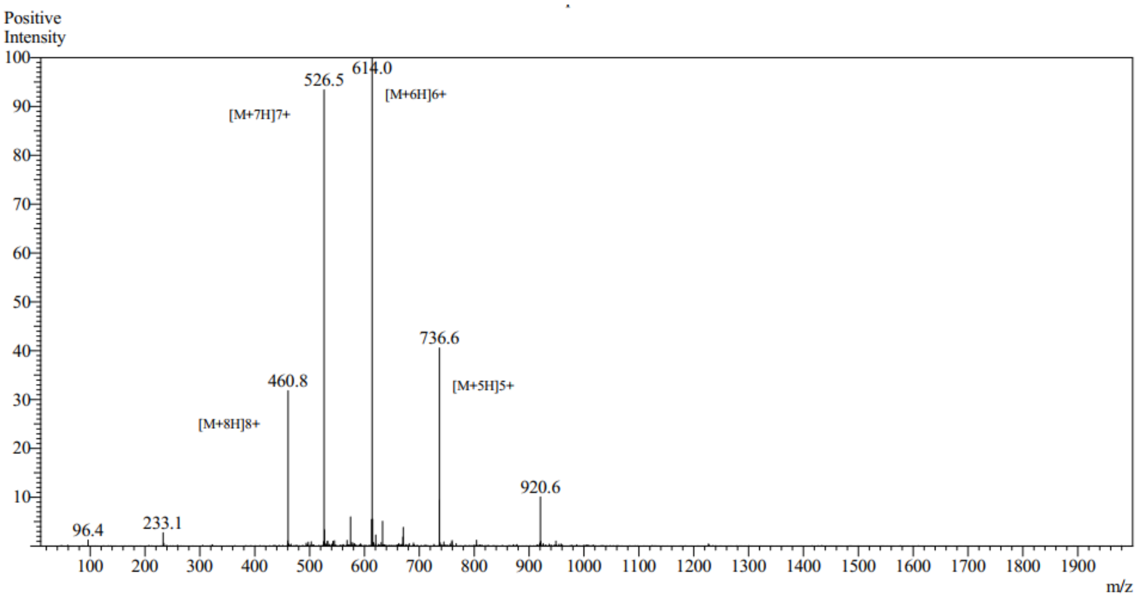

Fig. S2. Mass Spectrum for omCK11-31

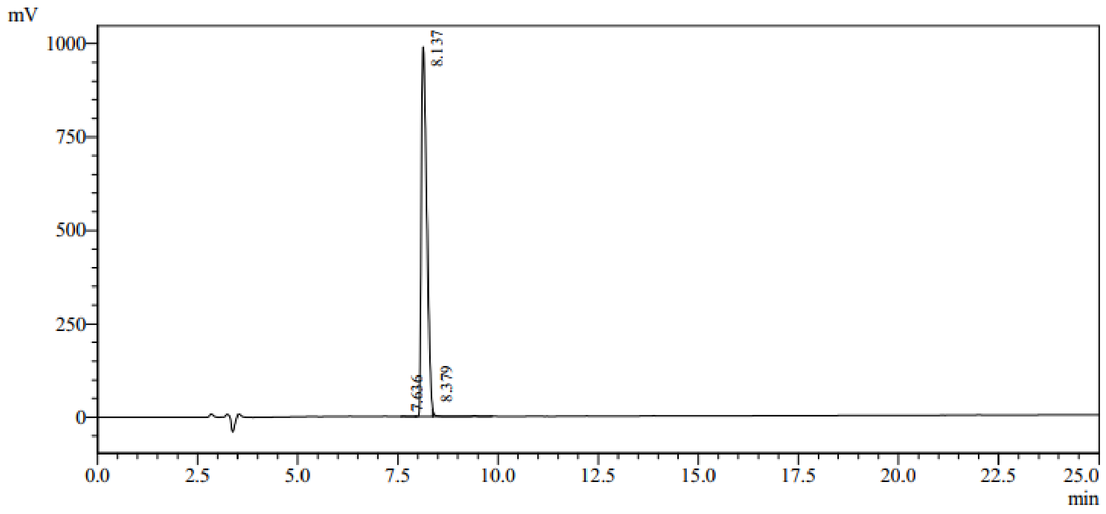

Fig. S3. Analytical HPLC for omCCL28-like-23

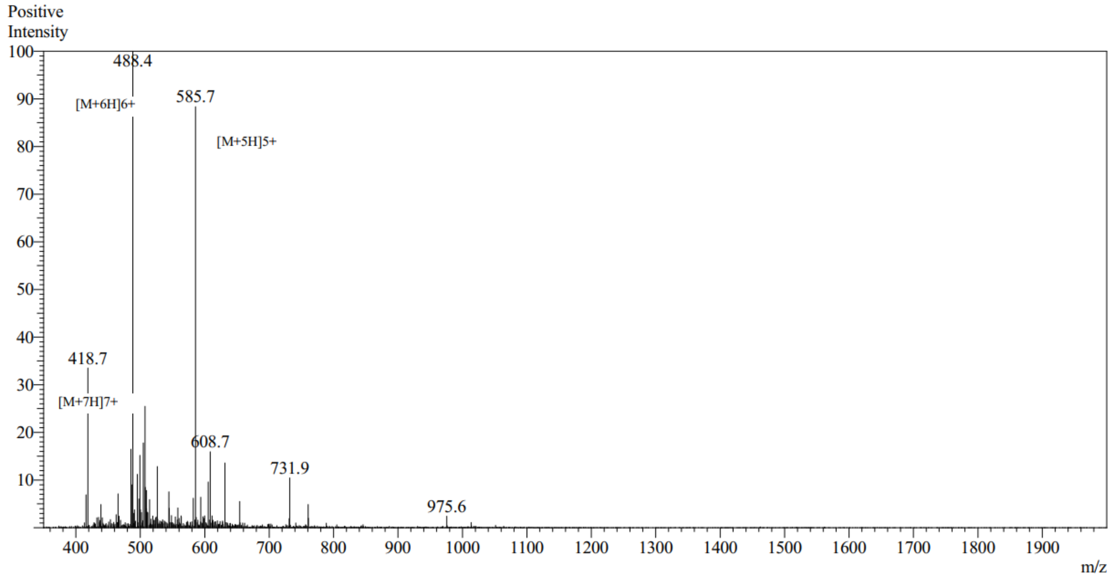

Fig. S4. Mass Spectrum for omCCL28-like-23

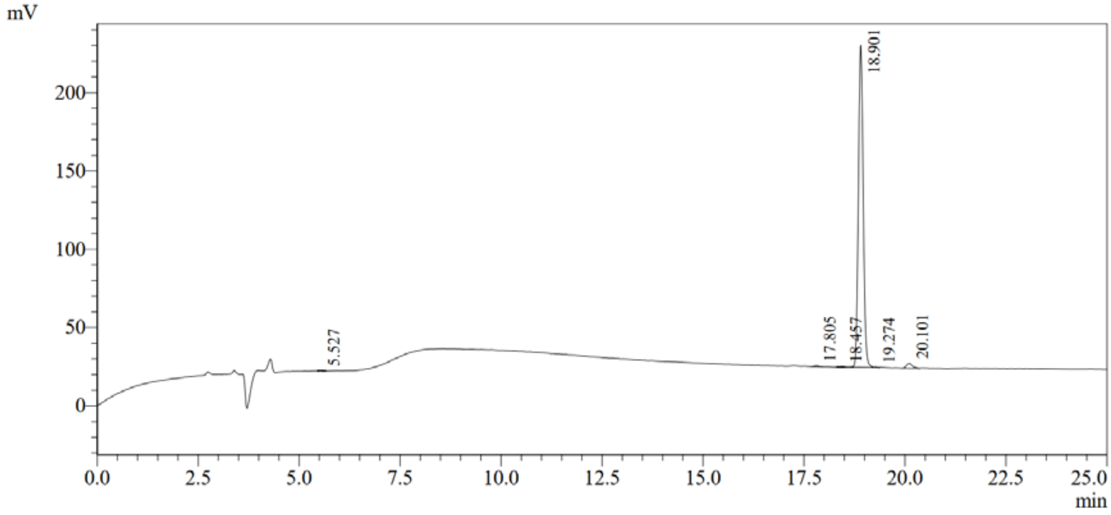

Fig. S5. Analytical HPLC for drCCL27b-24

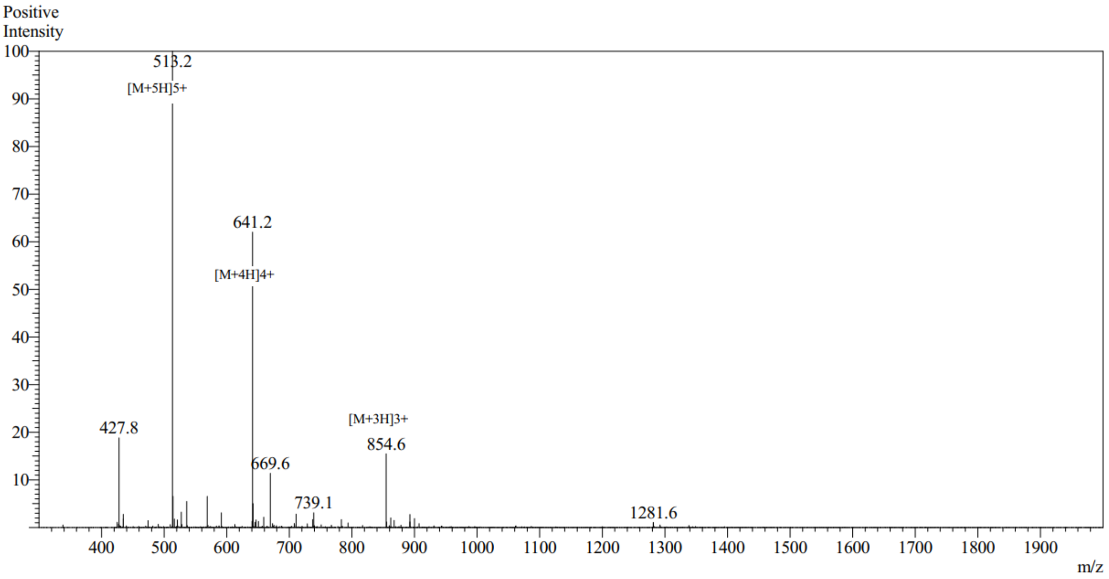

Fig. S6. Mass Spectrum for drCCL27b-24

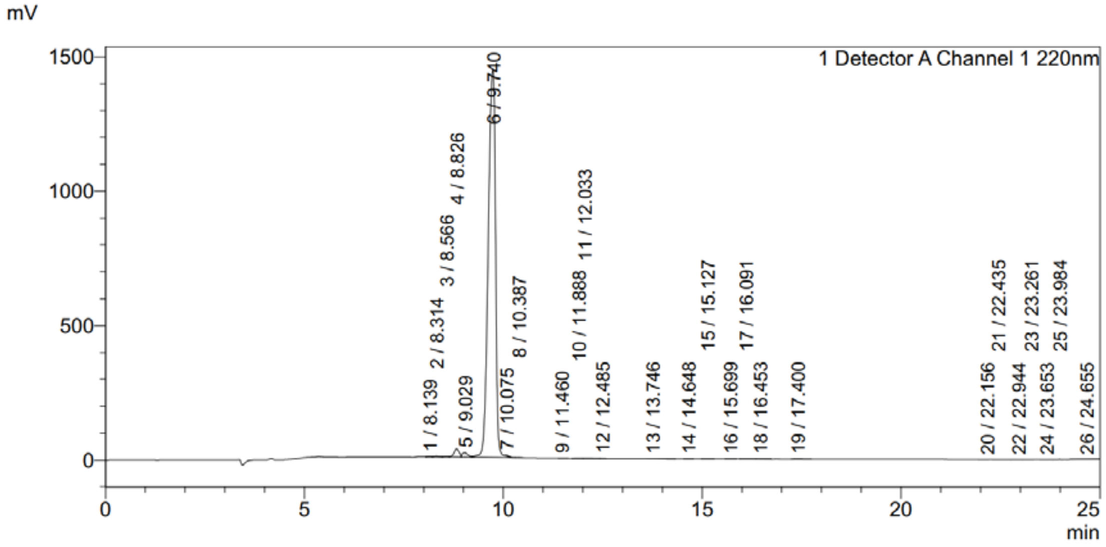

Fig. S7 Analytical HPLC for lcCCL28-25

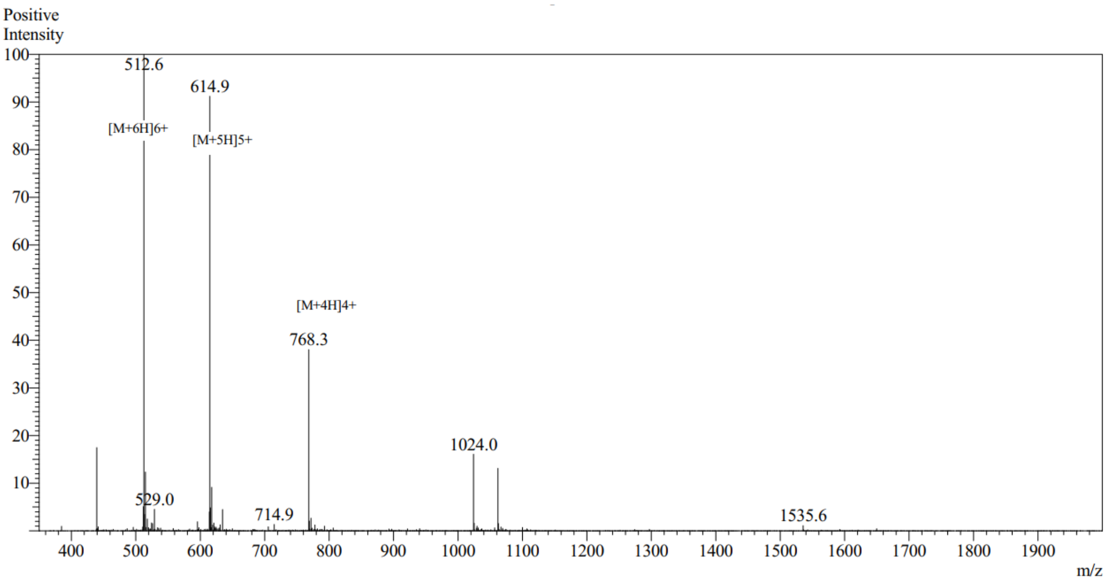

Fig. S8. Mass Spectrum for lcCCL28-25

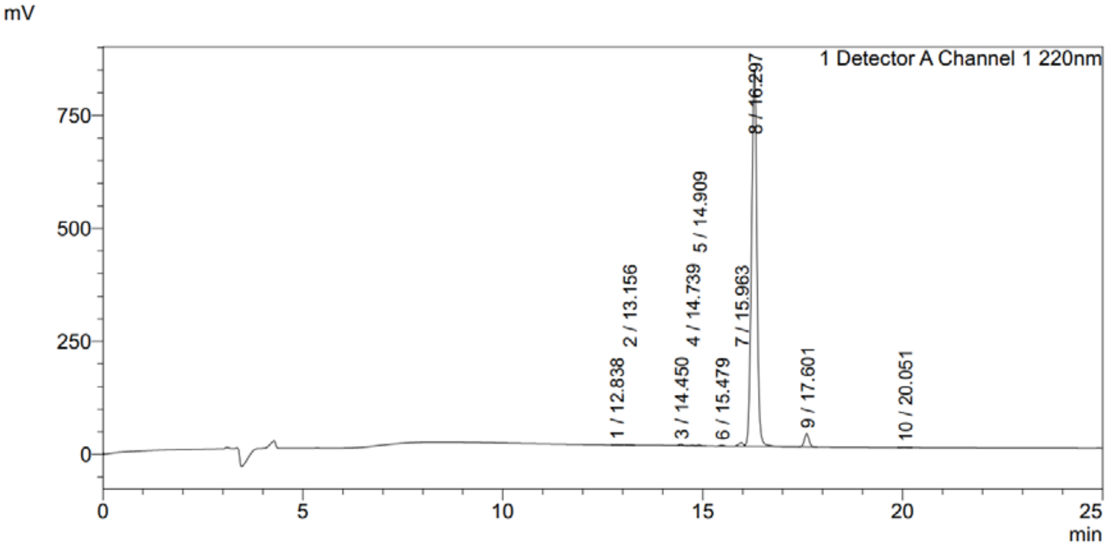

Fig. S9. Analytical HPLC for smCCL27b-25

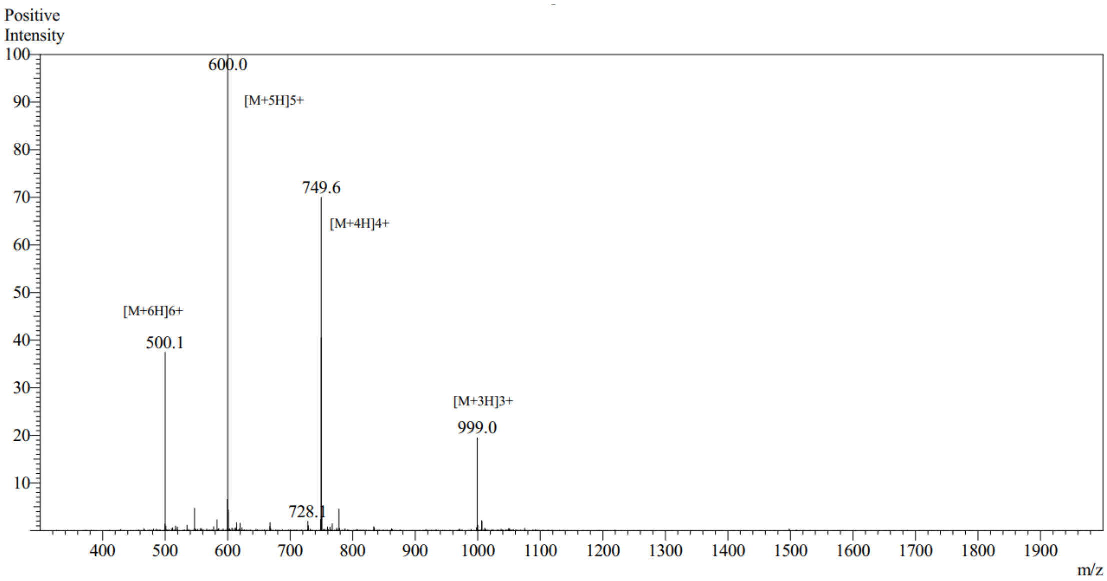

Fig. S10. Mass Spectrum for smCCL27b-25

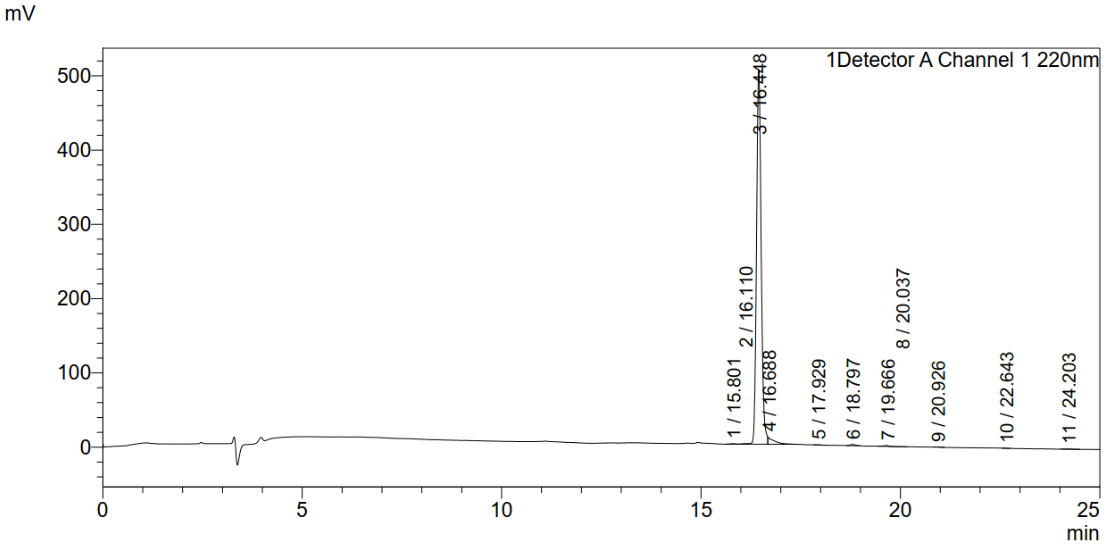

Fig. S11. Analytical HPLC for trCCL28-29

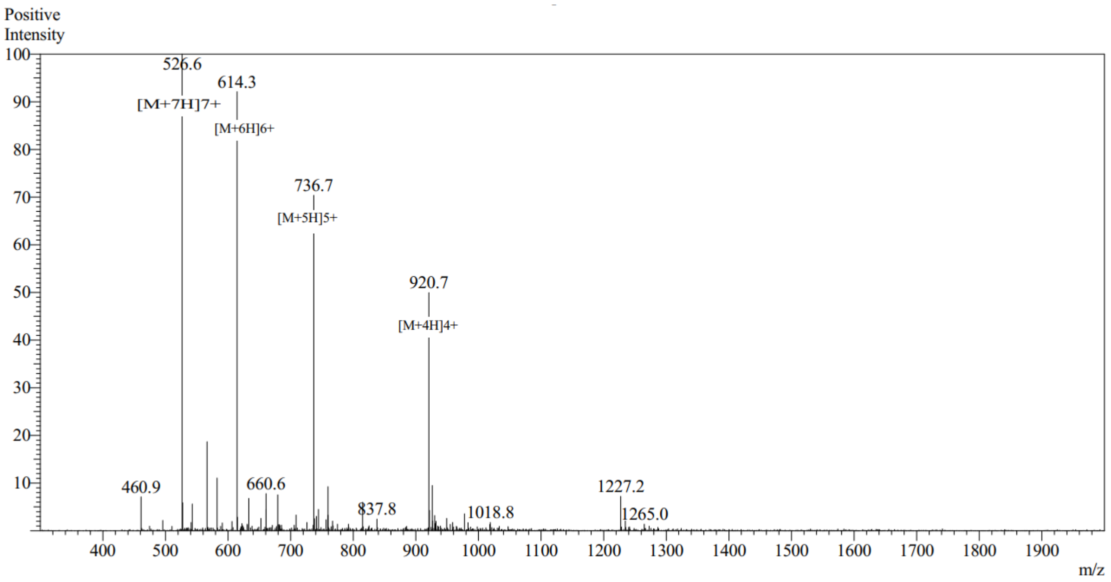

Fig. S12. Mass Spectrum for trCCL28-29

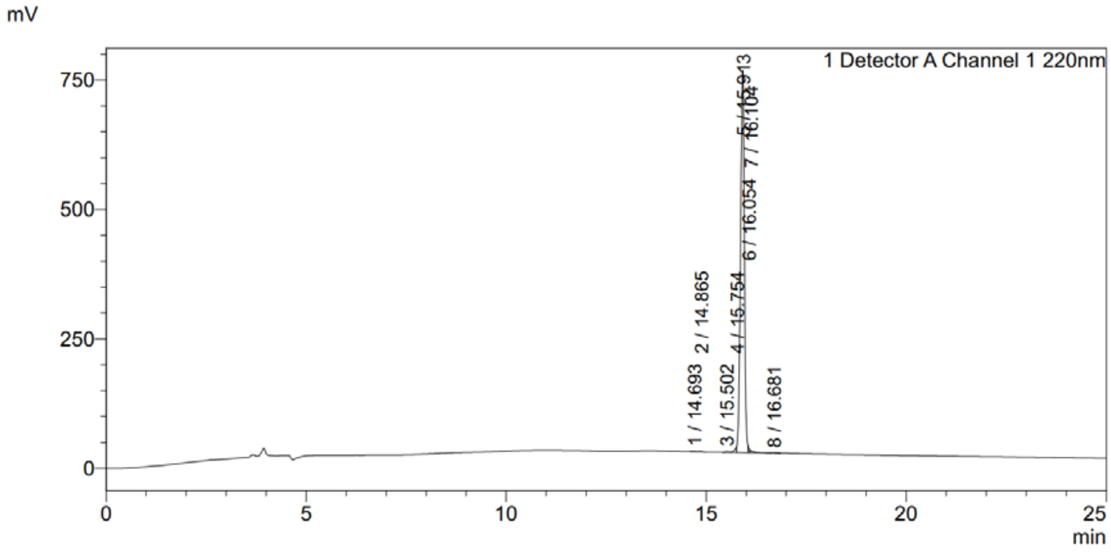

Fig. S13. Analytical HPLC for chCCL27a-26

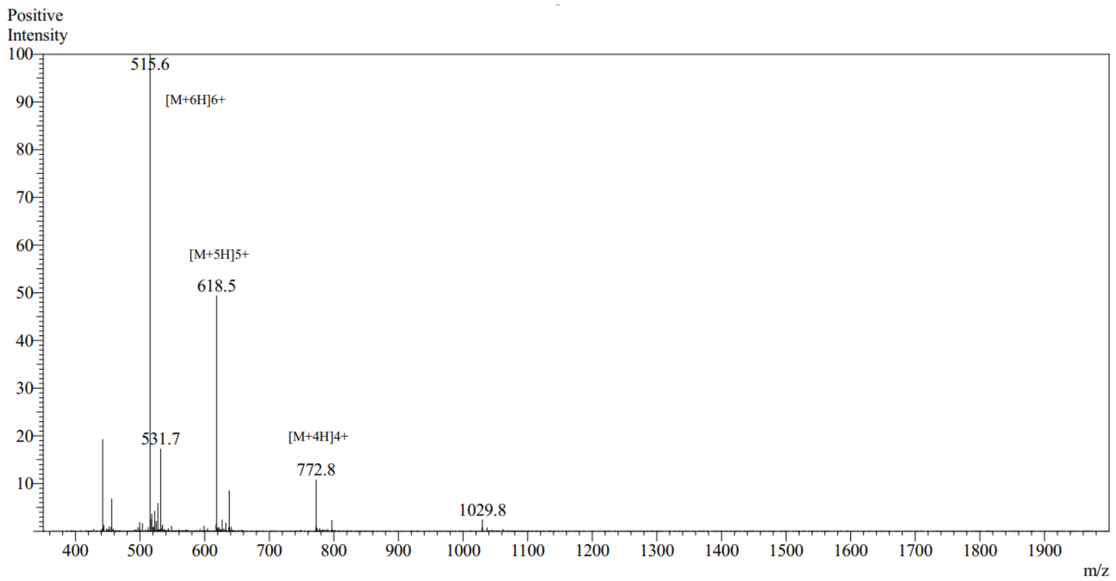

Fig. S14. Mass Spectrum for chCCL27a-26

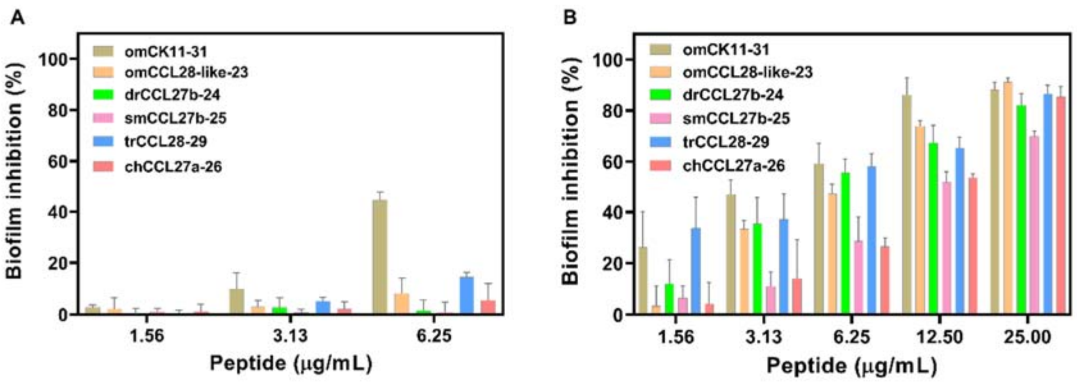

Fig. S15 Inhibition **(A)** and eradication **(B)** effect of peptides on *P. aeruginosa* FRD1 biofilm. 0% inhibition/eradication was established without peptide. Each measurement was performed in triplicate.

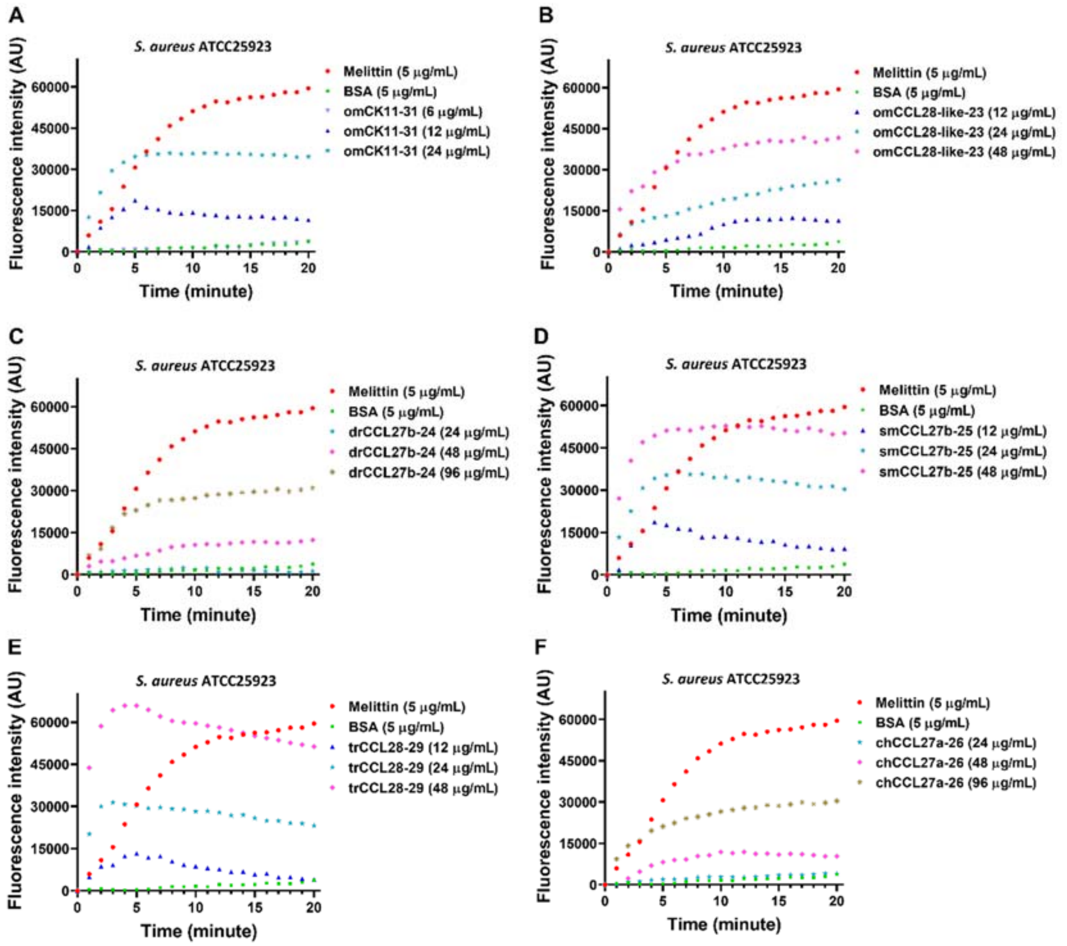

Fig. S16 Effects of peptides on cell membrane permeability of *S. aureus* ATCC25923. **(A)** omCK11-31. **(B)** omCCL28-like-23. **(C)** drCCL27b-24. **(D)** smCCL27b-25. **(E)** trCCL28-29. **(F)** chCCL27a-26. Bacterial ingestion of SYTOX Green was quantified by measuring intracellular fluorescence every 1 minute.

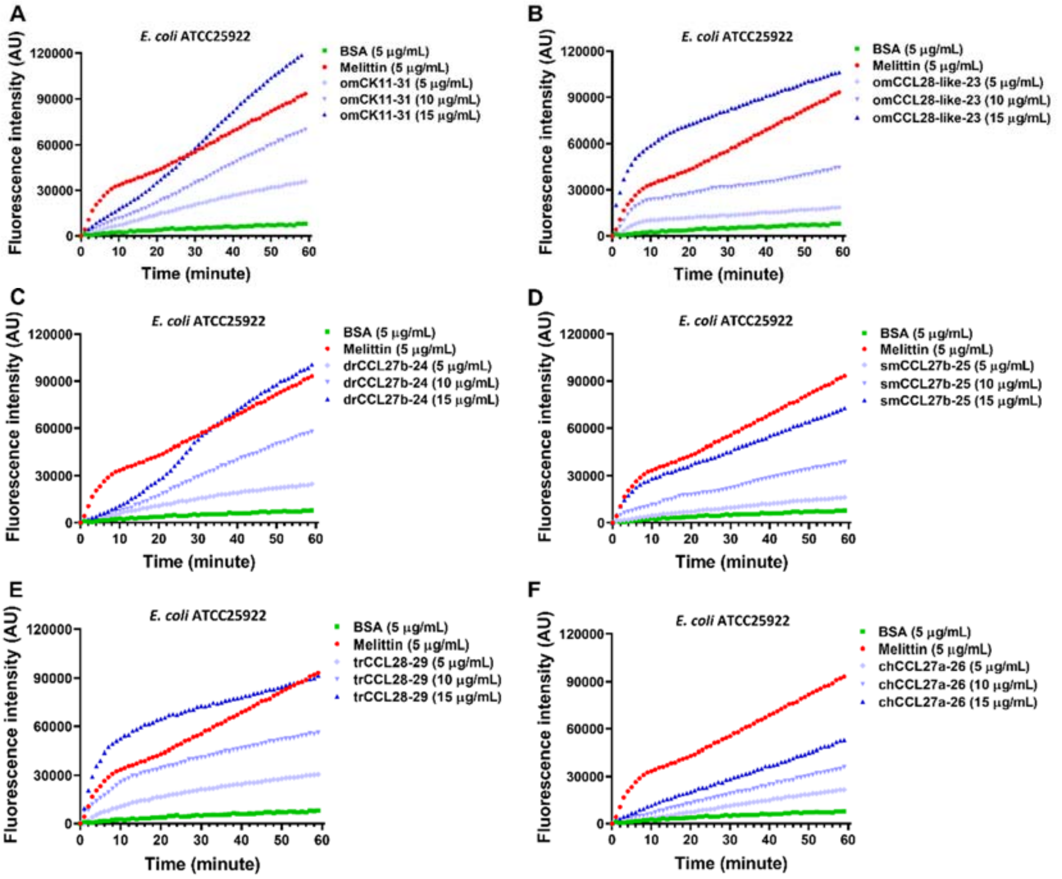

Fig. S17 Effects of peptides on cell membrane permeability of *E. coli* ATCC25922. **(A)** omCK11-31. **(B)** omCCL28-like-23. **(C)** drCCL27b-24. **(D)** smCCL27b-25. **(E)** trCCL28-29. **(F)** chCCL27a-26. Bacterial ingestion of SYTOX Green was quantified by measuring intracellular fluorescence every 1 minute.
